# Novel Mitogenome of *Garra manipurensis* Reveals Gene Rearrangement, Purifying Selection, and Matrilineal Phylogenetic Insights in Garrini (Cypriniformes: Cyprinidae)

**DOI:** 10.3390/ijms27125555

**Published:** 2026-06-19

**Authors:** Bungdon Shangningam, Angkasa Putra, Thonbamliu Abonmai, Agus Mohammad Hikam, Paya Torisha, Hyun-Woo Kim, Kyoungmi Kang, Shantanu Kundu

**Affiliations:** 1 Zoology Department, Ghanapriya Women College, Dhanamanjuri University, Imphal 795001, India; 2 Interdisciplinary Program of Marine and Fisheries Sciences and Convergent Technology, Pukyong National University, Busan 48513, Republic of Korea; 3 Department of Marine Biology, College of Fisheries Science, Pukyong National University, Busan 48513, Republic of Korea; 4 Marine Integrated Biomedical Technology Center, National Key Research Institutes in Universities, Pukyong National University, Busan 48513, Republic of Korea; 5 Research Center for Marine Integrated Bionics Technology, Pukyong National University, Busan 48513, Republic of Korea; 6 Department of Biology, Faculty of Science and Technology, Airlangga University, Surabaya 60115, Indonesia; 7 Ocean and Fisheries Development International Cooperation Institute, College of Fisheries Science, Pukyong National University, Busan 48513, Republic of Korea; 8 International Graduate Program of Fisheries Science, Pukyong National University, Busan 48513, Republic of Korea

**Keywords:** freshwater fish, molecular systematics, mitochondrial DNA, evolution, haplotyping, Indo-Burma biodiversity hotspot

## Abstract

Prior to this study, knowledge on the evolutionary lineage of *Garra* remained inadequate, as previous phylogenetic investigations were primarily based on partial gene sequences. Although several mitogenomes of *Garra* species have been reported, their structural organization and comprehensive genomic characteristics have not been thoroughly evaluated. In this study, *Garra manipurensis*, endemic to the Indo-Burma biodiversity hotspot, was identified based on its detailed morphology and meristic counts. The circular mitogenome of *G. manipurensis* is 16,776 bp in length and contains the canonical set of 37 genes, along with duplicated control regions separated by tRNA-Proline. The comparative assessments across *Garra* species indicate predominantly conserved GTG start codons, occasional alternative ATA initiation codons, and incomplete stop codons. The selection pressure examinations within Garrini taxa reveal a purifying selection across all protein-coding genes. The control region comprises four conserved sequence blocks and species-specific tandem repeats, reflecting a balance between functional constraint and lineage-dependent evolutionary dynamics. The phylogenetic inference supports the monophyly of *Garra* and places *G. manipurensis* in close affinity with *Garra flavatra*, which is native to the western slope of Rakhine Yoma in Myanmar and Mizoram State in northeastern India. The genetic diversity analyses revealed haplotype differentiation, with shallow intraspecific genetic distances (0.000–0.011) observed samples between two distinct drainage systems in Manipur and Mizoram, northeastern India. The observed pattern of haplotype divergence in *G. manipurensis* may reflect the historical or seasonal hydrological connectivity among the western-slope drainages of the Chin Hills, with the subsequent geographic isolation potentially contributing to the emergence of distinct genetic lineages. Nevertheless, the extent and evolutionary significance of this differentiation remain uncertain and warrant further investigation through expanded geographic sampling and the incorporation of additional molecular data. Collectively, these findings provide in-depth insights into the mitogenomic architecture, comparative gene arrangements, phylogenetic patterns, and matrilineal evolutionary history of *G. manipurensis* and other congeners, thereby improving our understanding of the systematics and genetic diversity of this important cyprinid fish lineage.

## 1. Introduction

Understanding teleost fish diversity represents a fundamental aspect of ichthyological research, providing essential insights into adaptive strategies, ecological functions, and evolutionary dynamics across diverse habitats [1,2]. In particular, freshwater fishes play crucial roles in maintaining ecosystem stability, promoting socio-economic development, supporting educational and conservation initiatives, and contributing to the sustainability of global food resources [3,4]. Among the various freshwater fish groups worldwide, the family Cyprinidae within the order Cypriniformes is recognized as one of the most species-rich lineages, marked by the extensive adaptive diversification that has enabled it to occupy a broad range of environmental conditions [5,6,7]. This family exhibits an almost cosmopolitan distribution, occurring on all continents except South America, Australia, and Antarctica, and currently comprises 1816 valid species classified into 168 genera and 10 subfamilies. Among them, the subfamily Labeoninae constitutes the largest group, encompassing 538 valid species distributed across 55 genera [7]. Within Labeoninae, the genus *Garra* represents the most diverse group, comprising small- to medium-sized benthic freshwater fishes that are predominantly algivorous and widely distributed across tropical and subtropical regions [8,9]. To date, *Garra* includes 195 valid species, with distributions extending across southern China, mainland and insular Southeast Asia, and South and West Asia, as well as eastern and western Africa [7]. From an economic perspective, several species within this genus are locally important as food resources and ornamental fishes, and in spa-based therapies, including fish pedicures and manicures, which have been used for the management of certain dermatological conditions [10]. Members of this genus are typically associated with upstream and midstream river sections characterized by fast-flowing waters, and mountainous stream systems in the Indo-Burma biodiversity hotspot [11,12]. Morphologically, members of the genus *Garra* are distinguished by an elongated and nearly cylindrical body shape, the presence of a serrated rostral fold on the upper lip, and a specialized gular disc structure on the lower lip, enabling their effective attachment to substrates in lotic freshwater ecosystems [13].

One of the major biogeographic centers of species diversity within the genus *Garra* is South Asia, where 98 valid species have been recorded, representing approximately 51% of all currently recognized species [7]. This remarkable species richness suggests that the region has contributed substantially to the diversification and evolutionary history of *Garra*. Among these species, *Garra manipurensis* is a benthopelagic taxon with a native distribution primarily confined to Manipur in northeastern India, with a distribution that may extend to the western slopes of the Rakhine Yoma Mountains in Myanmar [14,15,16]. Currently, this cyprinid species is listed as Vulnerable on the IUCN Red List of Threatened Species due to its limited distribution (<10,000 km^2^) and ongoing habitat degradation driven by dam construction, gravel and stone extraction, and fishing pressure [17]. Although the morphology of this species appears to be stable, an integrative systematic framework incorporating molecular genetic approaches is crucial for clarifying the systematics of *G. manipurensis*. Further, the molecular investigations of the genus *Garra* have increasingly employed mitochondrial fragments, nuclear loci, and microsatellite markers to evaluate the taxonomic boundaries, genetic diversity, population differentiation, and phylogenetic inferences [9,18,19]. However, most earlier studies were based on partial gene sequences and limited taxon sampling. In this context, the mitogenome represents a powerful resource for systematics and matrilineal evolutionary research due to its conserved genomic organization, high copy number, and relatively rapid mutation rate [20,21]. In vertebrates, the mitogenome is typically a circular DNA molecule of approximately 16–17 kb, comprising 13 protein-coding genes (PCGs), 22 transfer RNA (tRNA) genes, 2 ribosomal RNA (rRNA) genes, and a non-coding control region (CR) that regulates replication and transcription [20,21,22]. Accordingly, the mitochondrial data have been widely applied in phylogenetic construction and assessments of adaptive evolution across diverse cyprinid fishes [23,24]. Additionally, several models have been proposed to explain the mechanisms underlying mitochondrial gene rearrangements, including tandem duplication–random loss (TDRL) [25], recombination [26], tRNA mis-priming [27], tandem duplication–nonrandom loss (TDNL/RDNL) [28], and double replication–random loss (DRRL) [29]. Among these mechanisms, TDRL events appear to be the most frequently observed patterns of mitochondrial gene rearrangement in *Garra* species reported to date [8]. Consequently, characterizing the gene order of newly sequenced mitogenomes and elucidating the mechanisms underlying their rearrangements is essential for understanding the evolutionary processes and structural dynamics.

Despite these advances, complete mitogenomes are currently available for only a small fraction of recognized species within the genus *Garra*, representing approximately 7.7% of its known diversity (https://www.ncbi.nlm.nih.gov/nuccore/, accessed 15 June 2026). This limited genomic coverage constrains comprehensive matrilineal evolutionary inferences within *Garra*, particularly because comparative studies addressing the mitogenomic architecture and interspecific genetic variation remain scarce. Thus, to address the existing knowledge gaps, this study presents the first mitogenome of *G. manipurensis* from its native range in northeastern India and characterizes its genomic features in comparison with closely related congeners. The comparative mitogenomic examinations were conducted to explore the structural variation and gene arrangements, while phylogenetic inference was performed to clarify matrilineal relationships within the tribe Garrini. In addition, partial sequences of the mitochondrial cytochrome c oxidase subunit I (COI) gene were analyzed to further illuminate the genetic diversity across two drainage systems in northeastern India (Manipur and Mizoram). Overall, the integration of these approaches offers in-depth insights into the genomic profile, phylogenetic position, and genetic variability of *Garra* species, thereby providing a valuable framework for future systematic, evolutionary, and conservation studies.

## 2. Results

### 2.1. Morphological Characterization

**Figure 1:**
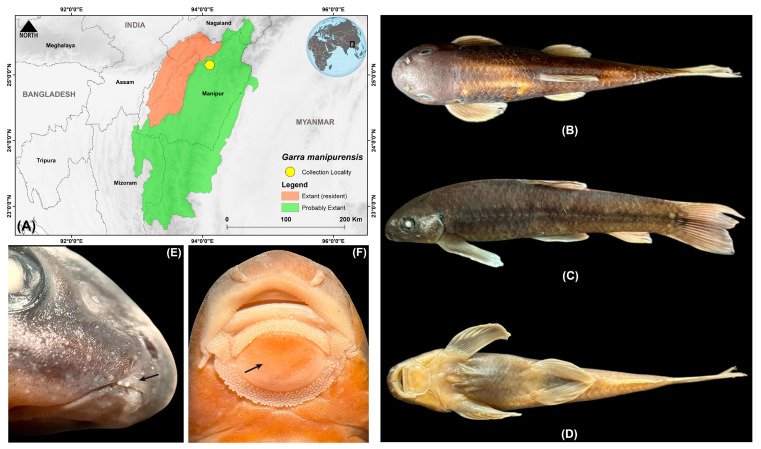
(**A**) The distribution range of the *G. manipurensis* in northeastern India and Myanmar, with the sampling locality in Manipur indicated by a yellow circle. (**B**–**D**) The morphological examination of a preserved specimen demonstrating dorsal, lateral, and ventral body perspectives. (**E**) The lateral view of the head highlighting rostral structure and orbital configuration, marked by black arrow. (**F**) The ventral view of the oral region illustrating the specialized scraping disc, denoted by black arrow. The fish photographs were taken by the first author (B.S.).

The examined specimens of *G. manipurensis* exhibited an elongated, cylindrical body and compressed laterally toward the caudal peduncle. The dorsal profile smoothly arched from the snout to the dorsal-fin origin, gradually sloping towards the caudal peduncle, the ventral profile from the pectoral-fin region to the caudal-fin origin, slightly convex (Figure 1B,C). Its head was relatively large, its width 20.3–22.0% SL and length 23.4–28.4% SL. Its head depth was 14.7–16.1% SL, depressed, with a slightly convex interorbital region (Table 1). The head was wider than its depth, with the head height less than its length. The eyes were comparatively large, positioned dorsolaterally, located closer to the posterior margin of the opercle than to the snout tip (Figure 1B–E). The snout was moderately rounded, and possessed a pair of rostral lobes covered with three to four small conical tubercles, with six to eight tubercles on each lobe. Minute conical tubercles were sparsely distributed on the snout tip, and internasal and interorbital regions with tiny pores and tubercles. The depressed rostral surface was flat. The sublachrymal groove was narrow and deep posteriorly, becoming wider anteriorly, extending dorsally along the base of the rostral barbel, surrounding it anteriorly, and continuing posteriorly to merge with the rostral cap groove. Two pairs of barbels were present. The rostral barbels originated anteroventrally, and were shorter than the eye diameter. The maxillary barbels were shorter than the rostral barbels, situated at the corners of the mouth. The rostral cap was short, well-developed, and moderately fimbriated, with a papillated ventral surface. The upper lip formed a thin band composed of weakly developed papillae arranged in a single row, uncovered by the rostral cap. The gular disc was elliptical, shorter than wide, narrower than the head (Figure 1B–F), located posteriorly, indicated by a transverse line through the base of the maxillary barbels crossing above the mid-length of the disc. The disc was relatively small, with a width of 43.1–46.8% HL and length of 28.5–37.1% HL, expanding anteriorly to the base of the lower jaw (Table 1). The torus of the mental adhesive disc was slightly curved, with lateral extensions reaching slightly beyond an imaginary vertical line drawn through the lateral margin of the pulvinus. The pulvinus was relatively small, measuring 31.7–38.0% HL in width and 17.6–22.9% HL in length, with the posterior portion more rounded than the anterior region (Table 1). The labellum was convex with a distinct posterior margin, with its upper marginal region partially covered by the rostral cap. The anterior margin of the labrum was extended to the level of the posterior margin of the eye, the groove separating the torus, with the pulvinus narrow and shallow. The anterior portion of the pulvinus contained a narrow papillated transverse lobe, demarcated posteriorly by a shallow transverse groove, separated anteriorly from the torus by another transverse groove. The papillae were concentrated on the ventral surface of the rostral cap, torus, labellum, and labrum, rounded and evenly arranged (Figure 1B–F).

There is a dorsal fin with two simple rays and 7½ branched rays, with the last simple ray shorter than the head length. The dorsal-fin origin is located midway between the snout tip and the caudal-fin base, aligned vertically with the pelvic-fin origin. The first branched ray is the longest, while the last branched ray is extended vertically to the level of the anal-fin origin. There is a pectoral fin with one simple and 12 branched rays, persisting beyond the midpoint between the pectoral-fin insertion and the pelvic-fin origin when adpressed. The sixth branched ray is the longest, shorter than the head length, with the fin margin appearing subacuminate. The pelvic fin possessed one simple and eight branched rays. The third branched ray is the longest, continuing beyond the midpoint toward the anal-fin origin and surpassing the anus. The anal fin is short with two simple rays and 3½ branched rays. The first branched ray is the longest, nearly reaching the caudal-fin base. The posterior margin of the anal fin is straight, the origin located midway between the pelvic-fin origin and the caudal-fin base. The anus is positioned posteriorly, with the distance from the anus to the anal fin representing 21.9–29.7% of the pelvic–anal distance. The caudal fin slightly emarginates with the pointed lobe tips with 10 + 11 principal rays. The lateral line is complete with 34 scales. The scale counts included 3½ transverse rows above and below the lateral line, circumpeduncular scales (16). The predorsal scales 13, regularly arranged. The chest and abdomen are scaled. A single axillary scale at the pelvic-fin base spanned to the posterior end of the pelvic-fin base. There are three preanal scales, with four scales at the dorsal-fin base, the last two connected to the fin base, whereas three scales occurred at the anal-fin base, with the last scale connected to the anal fin. The fresh specimens exhibited a brownish coloration on the head, dorsum, and lateral body surfaces. The dorsal, pectoral, pelvic, anal, and caudal fins showed an orange tinge. In the 10% preserved formalin, the head, dorsum, and lateral surfaces are dark greyish-brown, while the ventral mouth region, chest, and abdomen appeared yellowish. The fins have a greyish coloration after preservation. The posterior halves at the distal two-thirds of the dorsal-fin inter-radial membranes are black. A distinct black spot is present on the opercle immediately anterior to the upper angle of the gill opening. A faint greyish stripe continued along the lateral-line scales, across approximately half of scale rows above and below the lateral line, becoming more distinct posteriorly and prolonging onto the median rays of the caudal fin.

### 2.2. Mitogenomic Organization and Gene Arrangements

**Figure 2:**
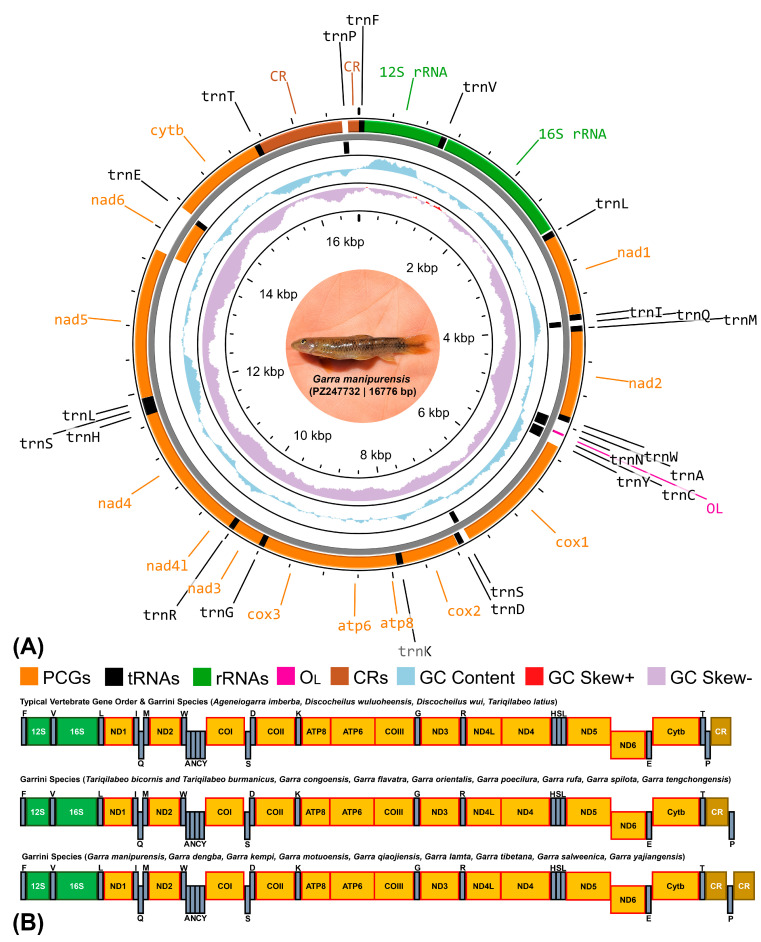
(**A**) The circular representation of the mitogenome architecture of *G. manipurensis*, with genes categorized using distinct color codes to facilitate structural interpretation. The fish photograph was taken by the first author (B.S.). (**B**) The linear representation of mitogenome gene arrangement among Garrini species with genes categorized using distinct color codes, highlighting variations in CR position. Multiple species exhibit CR relocation adjacent to tRNA-Pro, while *G. manipurensis* and related congeners possess a duplicated CR at the terminal region separated by tRNA-Pro.

**Figure 3:**
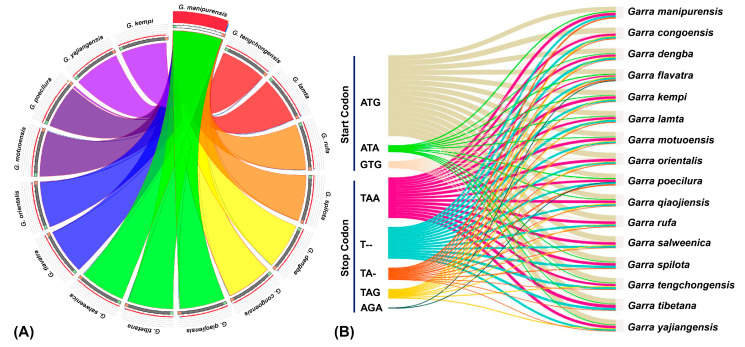
(**A**) The synteny-based homology analysis across 16 *Garra* mitogenomes. (**B**) The Sankey diagram depicting the frequency distribution of start and stop codons across the 13 PCGs of *G. manipurensis* and 15 other congeners.

The mitogenome of *G. manipurensis* was assembled as a circular DNA molecule with a total length of 16,776 bp and was deposited in GenBank under accession number PZ247732. It contained the typical complement of 37 mitochondrial genes, including 13 PCGs, 22 tRNAs, 2 rRNAs, 1 light-strand origin of replication (O_L_), and 2 CRs. Most genes were encoded on the heavy strand, whereas ND6 and eight tRNA genes were located on the light strand (Figure 2A; Table 2). The mitogenome exhibited a pronounced A+T bias, with adenine and thymine together accounting for 60.91% of the total nucleotide composition (A = 32.54%, T = 28.37%, G = 15.08%, C = 24.02%). The strand asymmetry analysis showed an AT-skew of 0.069 and a GC-skew of −0.229. The comparative examination across Garrini species indicated that the overall A+T content ranged from 57.23% in *Ageneiogarra imberba* to the highest value observed in *G. manipurensis*. The AT-skew values varied moderately among taxa, reaching up to 0.120 in *Garra salweenica* and *Garra spilota*, while the GC-skew values displayed limited variation, ranging from −0.274 in *G. salweenica* to −0.227 in *Garra rufa* (Table 3). Further, a total of 10 intergenic spacer regions (63 bp in total) and 12 overlapping regions (29 bp in total) were identified in the *G. manipurensis* mitogenome. The longest spacer (23 bp) was positioned between the 16S rRNA gene and tRNA-Leu2, whereas the longest overlaps (7 bp) were found at the ATP8–ATP6 and ND4L–ND4 junctions (Table 2). In comparison, most other *Garra* species presented their largest intergenic spacers between tRNA-Asp and COII, typically ranging from 7 to 13 bp (Table S2). In addition, the comparative mitogenomic assessment within Garrini revealed a conserved set of 37 genes and CR across all species examined, although variation in the number and arrangement of CRs was observed (Figure 2B). Four species (*A. imberba*, *Discocheilus wuluoheensis*, *Discocheilus wui*, and *Tariqilabeo latius*) reflected a linear mitogenome organization with the typical vertebrate gene order. In contrast, *Tariqilabeo bicornis*, *Tariqilabeo burmanicus*, and several *Garra* species (*G. congoensis*, *G. flavatra*, *G. orientalis*, *G. poecilura*, *G. rufa*, *G. spilota*, and *G. tengchongensis*) demonstrated minor rearrangements, characterized by the repositioning of the CR upstream of tRNA-Pro. Notably, *G. manipurensis* and eight other congeners (*G. dengba*, *G. kempi*, *G. motuoensis*, *G. qiaojiensis*, *G. lamta*, *G. tibetana*, *G. salweenica*, and *G. yajiangensis*) possessed a duplicated CR at the terminal region, separated by tRNA-Pro (Figure 2B). However, the comparative synteny investigation across all *Garra* sequences detected BLASTn e-values ranging between 11.20 and 181.00 (Figure 3A).

### 2.3. Features of Protein-Coding Genes

A total of 13 PCGs were identified in the mitogenome of *G. manipurensis*, spanning 11,399 bp and constituting ~67.93% of the entire mitogenome. The gene lengths varied considerably, with ATP8 representing the shortest PCG (165 bp) and ND5 exhibiting the greatest length (1821 bp) (Table 2). The canonical initiation using ATG predominated among PCGs; however, alternative start codons were also detected, including GTG in COI and ATA in both ND3 and ND5. Variability in the termination patterns was evident, as complete (TAA) and incomplete (TA- and T--) stop codons occurred among different genes. Specifically, TAA was found in ND1, ATP6, ATP8, ND4L, ND5, and ND6, whereas TA- was encountered in COI, COIII, and Cytb, while the remaining genes terminated with T-- (Figure 3B; Table 2). The comparative assessment across 16 other *Garra* species demonstrated that both start and stop codons remained generally conserved despite the moderate interspecific variation. The greater flexibility was observed in ND2, where ATA functioned as the initiation codon in both *G. flavatra* and *G. poecilura*. A similar variation was documented in ND5, for which ATA initiation was recorded in *G. dengba*, *G. kempi*, *G. lamta*, *G. motuoensis*, *G. qiaojiensis*, *G. spilota*, *G. tiengchongensis*, *G. tibetana*, and *G. yajiangensis*. The patterns of the stop codon also differed among *Garra* species. Although TAA persisted as the predominant termination codon, exceptions were confirmed in several taxa. Notably, ND5 ended with TAG in *G. rufa* and *G. salweenica*, contrasting with the TAA codon observed in other congeners. The stop codon of ND6 showed variation (AGA, TAG, or TAA), while the incomplete stop codons (T--) were recognized in COII, COIII, ND2, ND3, and ND4 across multiple *Garra* species (Figure 3B; Table S3).

### 2.4. Patterns of Nucleotide Substitution, Selective Pressure, and Codon Usage

**Figure 4:**
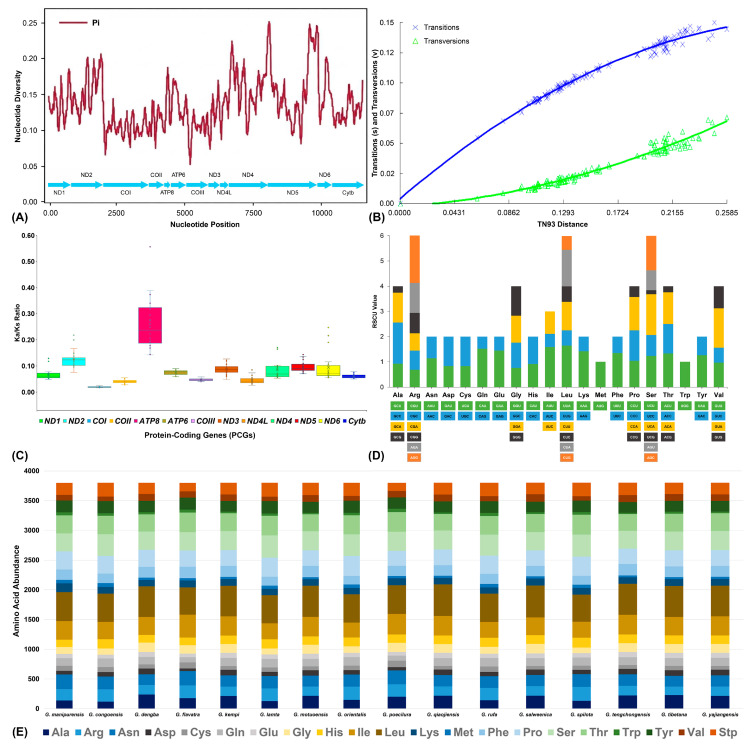
(**A**) The nucleotide diversity (π) across 13 PCGs among representative *Garra* species. (**B**) The relationship between transition–transversion substitution patterns and genetic divergence estimated using TN93 distance metrics. (**C**) The comparative analysis of Ka/Ks ratios across 13 PCGs of *G. manipurensis* and related congeners within tribe Garrini. (**D**) The RSCU profiles displaying codon preference and translational bias for individual amino acids in *Garra* species. (**E**) The comparative distribution of amino acid composition among examined *Garra* taxa.

The sliding-window analysis of mitochondrial PCGs across 16 *Garra* species identified substantial sequence variation, with 4775 polymorphic sites and nucleotide diversity (π) averaging 0.1339 (Figure 4A). Despite the observed variation, substitution saturation was not detected, as the increasing TN93 genetic distances suggested that sequence divergence accumulated proportionally among the examined taxa (Figure 4B). The selective constraint assessment based on Ka/Ks ratios revealed a purifying selection throughout PCGs in *G. manipurensis* and 21 representative Garrini species. The lowest evolutionary rate occurred in COI (0.0200 ± 0.0020), while ATP8 displayed the highest ratio (0.2610 ± 0.1000) (Figure 4C; Table S4). Across individual PCGs, the Ka/Ks values increased sequentially from COI to COII, ND4L, COIII, Cytb, ND1, ATP6, ND4, ND3, ND6, ND5, and ND2, with ATP8 representing the upper limit of the observed range. The patterns of synonymous codon utilization were uneven among PCGs, supporting the occurrence of codon usage bias. Although codon preference differed among taxa, translated protein lengths remained highly conserved, ranging only between 3799 and 3803 codons. Among all codons, GCG encoding alanine exhibited the lowest frequency of use. In *G. manipurensis*, codons characterized by RSCU values ≥ 1 constituted half of the total codon compilation, whereas those with values below one represented 45.31% (Figure 4D; Table S5). The profiling of the amino acid composition further demonstrated a disproportionate residue abundance, with arginine, leucine, and serine occurring most frequently. Their predominance may be associated with greater synonymous coding redundancy, as each is encoded by six alternative codons. Conversely, both methionine and tryptophan contributed minimally to the overall amino acid profile (Figure 4E; Table S6).

### 2.5. Characteristics of Ribosomal and Transfer RNA Genes

The mitogenome of the newly analyzed *G. manipurensis* comprised two rRNA genes and 22 tRNA genes (Table 2). The small ribosomal subunit gene (12S rRNA) was 942 bp in length, whereas the large ribosomal subunit gene (16S rRNA) measured 1650 bp, yielding a total rRNA length of 2592 bp and accounting for ~15.45% of the complete mitogenome. In contrast, the 22 tRNA genes collectively spanned 1566 bp, representing ~9.33% of the mitogenome (Table 2). The structural annotation confirmed the presence of all tRNA genes typically found in vertebrate mitogenomes. The identified tRNAs exhibited conserved structural domains, including the acceptor stem, dihydrouridine (DHU) arm, anticodon loop, and TΨC arm. The predicted secondary structures indicated that 21 tRNAs retained the canonical cloverleaf architecture characteristic of functional metazoan mitochondrial tRNAs, while the tRNA-Ser1 lacked the DHU arm (Figure S1). The stability of tRNA secondary structures was supported by both a canonical Watson–Crick base pairing (A–T and G–C) and non-canonical G–U wobble interactions. The G–U wobble pairing was observed in most detected tRNAs, including tRNA-Val, tRNA-Leu2, tRNA-Gln, tRNA-Met, tRNA-Ala, tRNA-Asn, tRNA-Cys, tRNA-Tyr, tRNA-Ser2, tRNA-Asp, tRNA-Arg, tRNA-Leu1, tRNA-Glu, tRNA-Thr, and tRNA-Pro (Figure S1).

### 2.6. Profiles of Non-Coding Control Region

**Figure 5:**
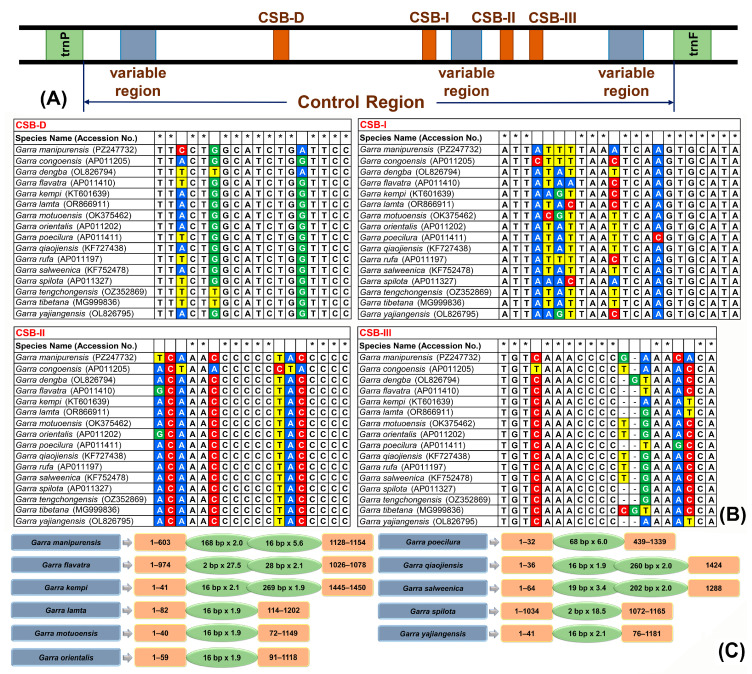
(**A**) The linear representation of the mitochondrial CR. (**B**) The nucleotide organization of CRs showing conserved sequence blocks (CSB-D, CSB-1, CSB-2, and CSB-3) and their corresponding lengths in *G. manipurensis* and 15 other *Garra* species. The conserved nucleotides shared among all analyzed species are indicated by asterisks on a white background, whereas the colored nucleotides represent variable positions across different species. (**C**) The species-specific tandem repeat motifs within the CR, where colored ovals reflect repeat units and copy number variations detected exclusively in 11 *Garra* species.

The CR of *G. manipurensis* was 1154 bp long, accounting for ~6.88% of the complete mitogenome (Table 2). The comparative assessment of CR sequences with 15 other *Garra* species identified four conserved sequence blocks (CSBs), namely, CSB-D, CSB-1, CSB-2, and CSB-3 (Figure 5A). These conserved elements were generally maintained among species; however, several nucleotide variations were observed, particularly within CSB-1, which represented the longest conserved segment (22 bp). In comparison, both CSB-D and CSB-2 had lengths of 18 bp, whereas CSB-3 extended to 20 bp. The sequence similarity analysis further indicated that CSB-D and CSB-1 were the most conserved regions, displaying values of 83.33% and 72.73%, respectively, while lower values were detected in CSB-3 (70.00%) and CSB-2 (61.11%) (Figure 5B). Moreover, the tandem repeat motifs within the CR were recorded in only 11 examined *Garra* species, whereas no repeat sequences were recognized in the remaining taxa. Among species containing tandem repeats, *G. manipurensis* together with *G. flavatra*, *G. kempi*, *G. qiaojiensis*, and *G. salweenica* exhibited a doubled repeat period length and copy number relative to the other species possessing single-copy motifs. A 16 bp repeat motif occurred approximately 1.9 times in *G. lamta*, *G. motuoensis,* and *G. orientalis*, while *G. yajiangensis* showed a slightly higher repeat frequency, reaching around 2.1 repeat units (Figure 5C).

### 2.7. Matrilineal Phylogenetic Placement and Genetic Divergence

**Figure 6:**
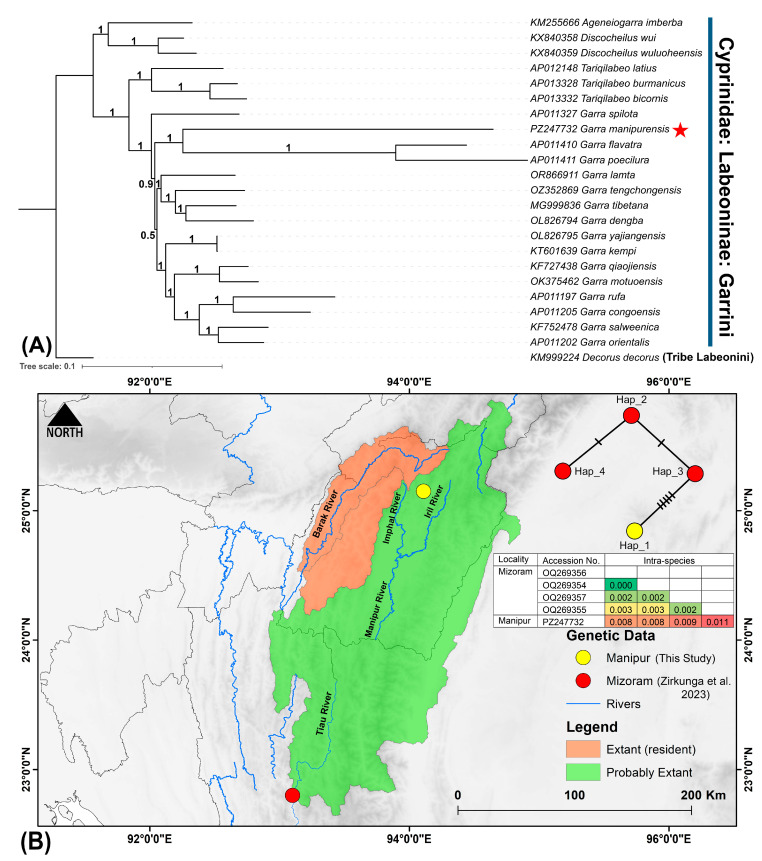
(**A**) The BA phylogenetic construction based on concatenated sequences of 13 PCGs, confirming monophyletic relationships among 16 *Garra* species within tribe Garrini. The posterior probability values are presented on branches and the *G. manipurensis* is marked with a red star symbol. (**B**) The map visualizing geographic distribution of *G. manipurensis* in Manipur and Mizoram, northeastern India, alongside a TCS haplotype network illustrating relationships among all haplotypes. The circle sizes are proportional to haplotype frequencies and hatch marks indicate mutational steps. A heatmap of pairwise genetic distances in K2P model suggests low genetic divergence between the two localities. [12].

The phylogenetic constructions generated using concatenated sequences of 13 PCGs under both BA and ML approaches consistently recovered representative species of the tribe Garrini according to their corresponding genera (Figure 6A and Figure S2). The resulting topologies strongly supported the monophyly of the *Garra* lineage and clarified the matrilineal evolutionary relationships among the investigated taxa. The inferred phylogenetic trees resolved two principal clades within the current dataset. One clade grouped *Ageneiogarra* and *Discocheilus*, genera mainly occurring in East Asia and mainland Southeast Asia. The second clade consisted of a strongly supported sister relationship between *Garra* and *Tariqilabeo*, with the latter genus distributed across East Asia, mainland Southeast Asia, and parts of South and West Asia (Figure 6A and Figure S2). Within the BA analysis, *G. manipurensis* exhibited a close phylogenetic association with *G. flavatra*, a species also distributed along the western slopes of the Rakhine Yoma in Myanmar and Mizoram in northeastern India. These two taxa also clustered together with *G. poecilura*, inhabiting the eastern slopes of the Rakhine Yoma and the Irrawaddy River basin in Myanmar (Figure 6A). Furthermore, the haplotype network assessment identified four distinct haplotypes of *G. manipurensis* across two sampling localities (Manipur and Mizoram). The genetic indices revealed a high haplotype diversity (Hd = 0.9000), accompanied by nucleotide diversity (π = 0.0047) and seven polymorphic sites (S). The genetic differentiation between the samples from the two localities remained low, with intra-specific distances ranging from 0.000 to 0.011 (Figure 6B; Table S7).

## 3. Discussion

The ichthyological research on mitogenomes within the genus *Garra* has expanded considerably over time; however, most studies have primarily concentrated on species identification and basic genomic characterization. Consequently, the signatures of structural variation and their evolutionary implications remain poorly resolved within this cyprinid group. To address this knowledge gap, the present study characterizes the mitogenome of morphologically identified *G. manipurensis* by integrating structural annotation with comparative genomic analyses across 15 other *Garra* species. Specifically, the mitogenome of *G. manipurensis* retains the standard vertebrate mitochondrial gene complement, consisting of 37 conserved genes. Within the non-coding regions, the origin of light-strand replication (O_L_) is situated in the WANCY tRNA cluster and is predicted to form a typical stem–loop secondary structure, a framework widely documented in teleost mitogenomes [8,20]. In addition, duplicated CRs were identified at the terminal portion of the mitogenome in *G. manipurensis*, a pattern also observed in eight other *Garra* species. This finding indicates notable structural variability within the genus and denotes the potential diversity in mitogenome organization [8,30]. The occurrence of duplicated CRs, together with their positional shift toward the tRNA-Pro region examined in this study, may provide evidence for a TDRL event, as similar gene rearrangements involving duplicated CRs have been reported previously [8,31]. Such TDRL-mediated rearrangements are thought to arise from the incomplete deletion of duplicated genomic segments following tandem duplication, resulting in the retention of intergenic spacer regions and/or pseudogenes [32,33]. These findings suggest that TDRL has played an important role in shaping the mitogenome architecture of *Garra* species. Such duplications have also been hypothesized to facilitate a role in modulating replication initiation and transcriptional regulation, which, in turn, could influence the mitochondrial copy number and gene expression dynamics [34]. Across the analyzed *Garra* species, the nucleotide composition exhibited a pronounced A+T bias accompanied by measurable AT and GC skews. This compositional pattern is consistent with asymmetric replication processes commonly recorded in fish mitogenomes, including those within the cyprinid lineage [8,20,21,23,24]. Furthermore, the comparative genomic assessment revealed the variation in the intergenic spacer length and gene overlap among species. In particular, extended overlaps between ATP8–ATP6 and ND4L–ND4 demonstrate a conserved genomic organization, likely displaying structural and functional constraints affecting the mitogenome configuration [23]. The ATG consistently served as the dominant initiation codon throughout the genus, whereas the GTG in COI appeared to be a conserved feature among studied species. This profile corresponds to typical teleost mitogenomes and reflects the preserved flexibility in mitochondrial translation [35]. Conversely, the presence of incomplete stop codons across *Garra* mitogenomes supports a well-established post-transcriptional completion mechanism, in which polyadenylation restores functional termination codons during mRNA processing [36,37].

The detected nucleotide diversity, together with the absence of substitution saturation in *Garra* PCGs, indicates that these genes retain a sufficient phylogenetic signal suitable for evolutionary and population genetic examinations, consistent with previous fish mitogenomic studies [38]. Analyses of the selective pressure showed that all 13 PCGs in Garrini taxa consistently display Ka/Ks ratios below ‘1’, indicating the purifying selection driven by strong functional constraints on mitochondrial energy metabolism [39]. Among them, COI demonstrated low Ka/Ks ratios, implying a strong negative selection against deleterious amino acid substitutions. In comparison, ATP8 exhibited relatively higher Ka/Ks values, highlighting relatively relaxed selective constraints or lower functional limitations on amino acid substitutions [40]. Although all Ka/Ks values suggested purifying selection, the relatively low genetic divergence among Garrini species may influence the reliability of Ka/Ks estimates and the inference of selective pressures [35]. Therefore, future studies incorporating broader taxonomic sampling and nuclear genomic data may further improve our understanding of selective pressures within this group. Moreover, the codon usage bias was also evident in *Garra* mitogenomes, characterized by the uneven representation of synonymous codons. Such patterns likely arise from underlying nucleotide compositional bias combined with selective forces favoring translational efficiency and accuracy within the mitochondrial system [41]. In *G. manipurensis*, most tRNAs maintained the canonical cloverleaf structure, except for tRNA-Ser1, which exhibited a reduced DHU arm, a feature frequently reported in cyprinid mitogenomes [23,24,42]. In addition, the presence of the G–U wobble base pairing may enhance structural flexibility in tRNA molecules, potentially broadening the codon recognition capacity and improving the efficiency of mitochondrial translation in this cyprinid taxon [43]. Simultaneously, four CSBs were identified in the CR alongside variable tandem repeat motifs across several *Garra* species, signaling a dual evolutionary pattern in which conserved elements are preserved for essential roles in replication and transcription, while adjacent variable regions facilitate interspecific divergence [8,23,44]. In particular, the conserved domains CSB-1 to CSB-3 are mainly associated with replication initiation and transcriptional regulation, whereas CSB-D, which is typically related to the Extended Termination-Associated Sequences (ETAS) region, may additionally participate in replication termination and contribute to regulatory stability in the *Garra* taxa [38]. Furthermore, the tandem repeat motifs recognized in 11 *Garra* species may act as a source of mitochondrial length variation and potentially facilitate DNA looping structures involved in replication regulation [44,45]. Alterations in the repeat copy number and periodicity increase the plasticity of this non-coding region, possibly contributing to adaptive evolutionary processes among the studied species [46].

The present phylogenetic inference of the Garrini species provides a higher-resolution understanding of evolutionary relationships and minimizes the limitations commonly associated with analyses based on partial genetic markers [8,20,21,23]. In this regard, the mitogenome-based BA and ML approaches conducted in this study consistently recover the monophyly of the *Garra* lineage, thereby reinforcing earlier hypotheses derived from matrilineal phylogenetic frameworks [47,48,49,50]. Within this phylogenetic context, *G. manipurensis*, which is distributed across Manipur in northeastern India and the western slopes of the Rakhine Yoma in Myanmar, is closely related to *G. flavatra*, a species that occupies a similar geographic range. This affinity may reflect an evolutionary signal driven by historical biogeographic continuity and reveals multiple cross-drainage dispersals in South and Southeast Asia [9]. Similar patterns have also been reported in numerous cyprinid lineages and other fish groups, where closely related taxa exhibit overlapping distributions within the same or historically connected drainage systems [22,23,24,37,51]. Nevertheless, given the potential influence of gene mixing, hybridization, introgression, and incomplete lineage sorting within *Garra*, the phylogenetic trees illustrated in this investigation demonstrate an exclusively maternal evolutionary history [52]. Therefore, integrating both mitochondrial and nuclear genomic data in future studies will offer a more comprehensive insight into the species relationships and evolutionary history within this complex genus.

Moreover, the genetic diversity analysis of *G. manipurensis* revealed the presence of four distinct haplotypes across the Manipur and Mizoram localities, with no shared haplotypes between these two regions. Despite this geographic segregation, the haplotypes differed by only a small number of mutational steps and exhibited low intraspecific genetic distances, indicating shallow genetic divergence. This pattern suggests the emergence of genetic structure in *G. manipurensis*, likely shaped by the restricted gene flow among river systems and prolonged geographic isolation in northeastern India. Notably, the sequences generated from Mizoram (OQ269354–OQ269357) originated from a single locality in the Tiau River (22.80° N, 93.10° E), an international transboundary river between northeastern India and Myanmar that subsequently joins the Kaladan River system [12]. In contrast, the Manipur specimen (PZ247732) was collected from the Manipur River system, which flows southward into Chin State, Myanmar, merges with the Myittha River, and ultimately drains into the Chindwin River basin. Although these river systems are not directly connected under present-day hydrological conditions, the occurrence of distinct haplotypes within *G. manipurensis* may be explained by two non-mutually exclusive hypotheses. The observed genetic differentiation may reflect the historical isolation associated with geographically separated drainage systems. Both river systems belong to the Indo–Burma Mountain Belt, where tectonic evolution and paleo-drainage rearrangements may have created historical hydrological connections [53,54], thereby facilitating the dispersal and genetic exchange among ancestral lineages of *G. manipurensis*. Further, the seasonal flooding events and temporary stream connectivity along the western slopes of the Chin Hills may intermittently link the Kaladan and Manipur River systems, potentially allowing a limited contemporary gene flow. Such processes could contribute to the retention of a shared ancestry while simultaneously promoting a gradual genetic divergence across geographically isolated localities. Collectively, the detected haplotype differentiation likely reflects the combined influence of historical drainage connectivity, intermittent contemporary dispersal, and long-term geographic isolation, ultimately contributing to genetic structuring within *G. manipurensis*. Nonetheless, the limited number of samples and geographic coverage of the present study restrict the robustness of the inferred patterns of genetic differentiation at the population level. Therefore, these findings should be considered preliminary and require further validation through expanded sampling across a broader geographic range and multiple drainage systems, coupled with the generation of additional molecular data to rigorously test the hypotheses proposed in this study.

## 4. Materials and Methods

### 4.1. Taxon Sampling, Morphological Assessment, and Ethical Approval

A total of five specimens (male = 3; female = 2) of *G. manipurensis* were collected from the Barak–Meghna River drainage system, specifically from the Barak River near Machengluang, Luangdi Pabram, and Chiuluan villages in Tamenglong District, Manipur, India (25.14999° N, 94.10881° E; Figure 1A). The sampling was carried out independently by the authors using scoop nets during field surveys. After transportation to the laboratory, all specimens were anesthetized with 2-phenoxyethanol (600 µL L^−1^) until cessation of opercular movement was observed, followed by three rinses with Milli-Q water to remove residual anesthetic compounds [55]. The preserved voucher specimens were fixed in 10% neutral buffered formalin and deposited in the Department of Zoology, Dhanamanjuri University, India, under voucher numbers GWCDU-PKNU-LIFE-066 to GWCDU-PKNU-LIFE-070. The morphometric measurements were obtained using a dial caliper with an accuracy of 0.1 mm. Both measurements procedures and proportional analyses were performed according to methods previously described [56], with the inclusion of maximum body depth measured at the dorsal-fin origin. Head length (HL) and other morphometric traits were expressed as percentages of standard length (%SL), whereas cranial measurements were standardized relative to HL. Conversely, the fin-ray counts were determined using a stereo-zoom binocular microscope. For molecular analysis, ~150 mg of epaxial fresh muscle tissue was aseptically excised from a representative specimen in the region between the dorsal fin and lateral line before formalin fixation and voucher preservation. The tissue sample was stored in 95% ethanol in sterile 2 mL microtubes and subsequently maintained at −20 °C prior to DNA extraction to minimize degradation and contamination risks. Additionally, the biogeographical distribution of *G. manipurensis* throughout northeastern India and Myanmar was mapped using ArcGIS Pro v3.0 based on IUCN distribution shapefiles (Figure 1A). All experimental procedures received approval from the Animal Ethical Committee of Dhanamanjuri University, Imphal, India (No. 21/2024/DMU-LIFE/E.11, dated 10 December 2025), and complied with ARRIVE 2.0 guidelines for animal research (https://arriveguidelines.org, accessed on 1 May 2026) [57].

### 4.2. DNA Extraction, Library Preparation, and Mitogenome Sequencing

The mitochondrial DNA was isolated from ~30 mg of tissue of *G. manipurensis* using the Alexgen DNA Kit (Alexius Biosciences, Ahmedabad, Gujarat, India) following the manufacturer’s instructions [58]. The DNA concentration was thereafter quantified with a Qubit 4.0 fluorometer (Thermo Fisher Scientific, Waltham, MA, USA) to verify suitability for subsequent library preparation procedures. The sequencing was performed at the Neuberg Center for Genomic Medicine, Neuberg Supratech Research Laboratories (NSRL), Ahmedabad, India. A paired-end (2 × 150 bp) with a duplication rate of 0.864075% and an insert size peak of 218 bp sequencing libraries were generated using the QIAseq FX DNA Library Kit (Cat. No. 180479, Qiagen, Hilden, Germany). The genomic DNA was fragmented using a Covaris M220 Focused Ultrasonicator (Covaris Inc., San Diego, CA, USA) to obtain fragments of appropriate size for sequencing. The adapter ligation was then conducted to facilitate compatibility with the Illumina platform. To improve sequence coverage from limited DNA input, the libraries were amplified using HiFi PCR Master Mix (Takara Bio Inc., Kusatsu, Shiga, Japan). Both library integrity and fragment-size profiles were assessed with a TapeStation 4150 system (Agilent Technologies, Santa Clara, CA, USA) employing High-Sensitivity D1000 ScreenTape. The libraries meeting the required quality thresholds were subsequently subjected to paired-end high-throughput sequencing on the Illumina NovaSeq 6000 platform (Illumina, San Diego, CA, USA).

### 4.3. Mitogenome Assembly, Annotation, and Data Submission

A total of 16.458062 million raw reads were obtained prior to quality filtering, with Q20 and Q30 values of 99.262294% and 97.280282%, respectively. After filtering, 16.168060 million high-quality paired-end reads were retained (Q20 = 99.391914%; Q30 = 97.657453%) and subsequently used for mitogenome construction and annotation of *G. manipurensis* in Geneious Prime v2023.0.138 [59]. To improve annotation reliability, the genomic features including gene boundaries, strand orientation, and positional arrangement were independently assessed using MITOS2 via the Galaxy web platform v2.1.10 (https://usegalaxy.eu, accessed on 1 May 2026) and MitoAnnotator implemented in the Mitofish server v2025.06 (https://mitofish.aori.u-tokyo.ac.jp/annotation/input, accessed on 1 May 2026) [60,61,62]. The predicted amino acid translations generated under the vertebrate mitochondrial genetic code were inspected using the Open Reading Frame Finder (https://www.ncbi.nlm.nih.gov/orffinder, accessed on 1 May 2026) to validate the PCGs [63]. In contrast, both tRNA and rRNA annotations were additionally checked for consistency through the Galaxy web platform v2.1.10. The curated mitogenome was subsequently archived in GenBank under accession number PZ247732.

### 4.4. Mitogenome Structure and Comparative Examinations

The mitogenome of *G. manipurensis* was annotated and graphically represented using the CGView server (https://proksee.ca, accessed on 1 May 2026) [64]. The base composition across 22 species within the Garrini lineage was quantified in MEGA v12, while the nucleotide strand asymmetry was assessed through AT-skew [(A − T)/(A + T)] and GC-skew [(G − C)/(G + C)] calculations following established procedures [65,66]. The comparative genomic analyses were conducted using the newly assembled mitogenome together with 15 publicly available *Garra* mitogenomes obtained from GenBank (https://www.ncbi.nlm.nih.gov/nuccore/, accessed on 1 May 2026) (Table S1). Both sequence similarity and genomic synteny were evaluated using Circoletto v25.03.23 with a nucleotide-BLAST 2.17.0 approach, and outputs were displayed in a circular format employing a seven-color gradient among the 16 *Garra* species (https://bat.infspire.org/circoletto/, accessed on 1 May 2026) [67]. Both intergenic spacer lengths and overlapping regions were manually examined in Microsoft Excel v2016. Start and stop codons of the 13 PCGs were identified using the ORF Finder, and Sankey diagrams generated in SankeyMATIC (https://sankeymatic.com/, accessed on 1 May 2026) were used to visualize codon initiation and termination patterns. The genetic variation among *Garra* species was estimated by calculating nucleotide diversity (π) in DnaSP v6.0 under a sliding-window framework (200 bp window size; 25 bp step interval) [68]. Both transition and transversion substitution patterns across codon positions were investigated using the Tamura–Nei (TN93) genetic distances as implemented in DAMBE v6 [69], whereas synonymous (Ks) and nonsynonymous (Ka) substitution rates were also computed in DnaSP v6.0 to infer selective constraints among Garrini taxa. The codon usage patterns, including Relative Synonymous Codon Usage (RSCU) and amino acid frequency distributions, were further analyzed using MEGA v12. Exclusively, the secondary structures of tRNA genes in *G. manipurensis* were predicted with ARAGORN v1.2.41 via the Galaxy platform v2.1.10 [70]. The conserved sequence signatures within the CR between tRNA-Pro and tRNA-Phe were recognized in 16 *Garra* species through CLUSTAL W alignments and interpreted based on previously described teleost CR domains [20,71]. The tandem repeat motifs within the complete CR were determined using Tandem Repeats Finder (https://tandem.bu.edu/trf/trf.html, accessed on 1 May 2026) and may act as useful marker for future population genetic applications [72].

### 4.5. Dataset Collection, Phylogenetic Inferences, and Genetic Diversity

The phylogenetic relationships among 22 mitogenomes representing the genera *Garra*, *Ageneiogarra*, *Discocheilus*, and *Tariqilabeo* within the tribe Garrini were constructed using a concatenated dataset of 13 PCGs (Table S1). The cyprinid *Decorus decorus* (GenBank accession no. KM999224) served as the outgroup [73]. The sequence alignment of the concatenated PCGs was carried out in iTaxoTools v0.1 using the MAFFT algorithm under default settings [74]. The evolutionary affinities were inferred under both Bayesian Inference (BA) and Maximum Likelihood (ML) frameworks. The model testing in MEGA v12 identified the ‘GTR+G+I’ substitution scheme as the best-fit model based on the lowest Bayesian Information Criterion (BIC) value [75]. The BA phylogenetic tree was executed in MrBayes v3.1.2 using a Metropolis-coupled Markov chain Monte Carlo (MCMC) approach with one cold and three heated chains (nst = 6). The analysis ran for one million generations with sampling every 100 generations, while the initial 25% of trees were excluded as burn-in. The convergence diagnostics relied on the average standard deviation of split frequencies (≤0.01) together with potential scale reduction factor values approaching 1 [76]. In parallel, the ML inference was performed in PhyML v3.0 under the same substitution model (http://www.atgc-montpellier.fr/phyml/usersguide.php, accessed on 1 May 2026) [77]. Resulting topologies from both BA and ML frameworks were visualized and annotated using iTOL v7 (https://itol.embl.de/, accessed on 1 May 2026) [78]. For genetic diversity assessment, the partial COI sequences derived from the newly assembled mitogenome were merged with four publicly available sequences (OQ269354 to OQ269357) retrieved from GenBank [12]. Alignment of all COI datasets was completed in MEGA v12 using CLUSTAL W, producing a final 642 bp dataset for downstream investigations. The mean intraspecific divergence was computed under the Kimura 2-parameter (K2P) model in MEGA v12. Haplotype number, haplotype diversity (Hd), nucleotide diversity (π), and polymorphic sites (S) were quantified using DnaSP v6.0. The haplotype relationships were illustrated through a Templeton, Crandall, and Sing (TCS) network implemented in POPART v1.7 [79,80].

## 5. Conclusions

This study illuminates an in-depth characterization of the complete mitogenome of morphologically identified *G. manipurensis* from its native range in northeastern India. The genome consists of a circular DNA molecule and exhibits a gene order that is largely conserved with those reported in eight other *Garra* species. The comparative analyses across the genus revealed differences in gene organization, codon usage patterns, and nucleotide composition, indicating the notable mitogenomic variability within *Garra*. The CR was found to contain four CSBs together with species-specific tandem repeat motifs, suggesting the presence of both functional regulatory elements and lineage-specific evolutionary signatures among *Garra* species. The matrilineal phylogenetic inference based on concatenated sequences of 13 PCGs supported the monophyly of the genus *Garra* and demonstrated a close evolutionary relationship between *G. manipurensis* and *G. flavatra*. Furthermore, the COI-based haplotype network analysis provided preliminarily evidence of genetic differentiation across the distribution range of *G. manipurensis*. However, a broader geographic coverage, increased sample sizes, and the incorporation of multiple genetic markers are required to achieve a more comprehensive understanding of the phylogeographic structure of this cyprinid species. Nevertheless, future studies should also expand mitogenomic resources to encompass the remaining 91.79% of *Garra* diversity that remains undocumented, thereby providing deeper insights into the evolutionary history and diversification patterns of this lineage.

## Figures and Tables

**Table 1:** The morphometric data of *G. manipurensis* (male = 3 and female = 2).

Parameter	Range	Mean	S.D.
Standard length (mm)	40.0–48.5	43.5	3.7
**% SL**			
Body depth	21.0–23.6	22.0	1.1
Head length	23.4–28.4	26.8	2.3
Head depth at nape	9.7–11.9	10.9	0.9
Head depth at eye	14.7–16.1	15.4	0.7
Head width	20.3–22.0	21.0	0.8
Body width at anal-fin origin	10.9–12.0	11.6	0.5
Body width at dorsal-fin origin	15.3–19.1	17.2	2.0
Caudal peduncle length	14.4–17.4	15.7	1.3
Caudal peduncle depth	11.5–14.6	13.5	1.4
Dorsal-fin base length	13.4–15.2	14.0	0.8
Dorsal-fin length	20.2–24.8	22.7	1.9
Pectoral-fin length	24.3–29.0	26.4	2.4
Pelvic-fin length	20.3–23.6	22.0	1.5
Anal-fin base length	7.9–13.2	9.9	2.4
Anal-fin length	18.3–22.3	20.2	2.1
Predorsal length	51.5–57.1	53.5	2.6
Prepectoral length	21.9–23.7	23.0	0.8
Prepelvic length	51.8–53.2	52.7	0.6
Preanus length	69.5–71.8	70.2	1.1
Preanal length	76.3–79.8	78.2	1.5
Pelvic-anal distance	23.0–26.2	24.3	1.4
Snout length	11.8–13.1	12.2	0.6
Eye diameter	5.0–6.0	5.5	0.4
Interorbital distance	13.1–14.8	14.0	0.8
**%** **Pelvic–anal distance**			
Distance from anus to anal fin	21.9–29.7	27.5	3.8
**%** **HL**			
Snout length	42.3–50.5	45.9	3.9
Eye diameter	18.6–21.5	20.7	1.4
Interorbital distance	50.4–55.9	52.5	2.4
Disc width	43.1–46.8	45.5	1.6
Disc length	28.5–37.1	31.7	3.9
Pulvinus width	31.7–38.0	34.8	2.6
Pulvinus length	17.6–22.9	19.0	2.6

**Table 2:** Annotated genes of the *G. manipurensis* mitogenome, showing gene boundaries, lengths, and intergenic nucleotides. ‘H’ and ‘L’ denote genes encoded on the heavy and light strands, respectively, and a dash (-) indicates an incomplete stop codon.

Gene	Start	End	Strand	Size (bp)	Intergenic Nucleotide	Anti-Codon	Start Codon	Stop Codon
tRNA-Phe	1	68	H	68	−1	GAA		
12S rRNA	68	1009	H	942	0			
tRNA-Val	1010	1081	H	72	18	TAC		
16S rRNA	1100	2749	H	1650	23			
tRNA-Leu	2773	2848	H	76	1	TAA		
ND1	2850	3824	H	975	3		ATG	TAA
tRNA-Ile	3828	3900	H	73	−3	GAT		
tRNA-Gln	3898	3969	L	72	0	TTG		
tRNA-Met	3970	4039	H	70	0	CAT		
ND2	4040	5084	H	1045	0		ATG	T--
tRNA-Trp	5085	5155	H	71	0	TCA		
tRNA-Ala	5156	5225	L	70	0	TGC		
tRNA-Asn	5226	5299	L	74	0	GTT		
O_L_	5300	5327	H	28	0			
tRNA-Cys	5328	5394	L	67	0	GCA		
tRNA-Tyr	5395	5462	L	68	1	GTA		
COI	5464	7010	H	1547	0		GTG	TA-
tRNA-Ser	7011	7081	L	71	2	TGA		
tRNA-Asp	7084	7154	H	71	8	GTC		
COII	7163	7853	H	691	−1		ATG	T--
tRNA-Lys	7853	7931	H	79	1	TTT		
ATP8	7933	8097	H	165	−7		ATG	TAA
ATP6	8091	8774	H	684	−1		ATG	TAA
COIII	8774	9558	H	785	−1		ATG	TA-
tRNA-Gly	9558	9629	H	72	0	TCC		
ND3	9630	9978	H	349	−1		ATA	T--
tRNA-Arg	9978	10,047	H	70	0	TCG		
ND4L	10,048	10,344	H	297	−7		ATG	TAA
ND4	10,338	11,718	H	1381	−1		ATG	T--
tRNA-His	11,718	11,787	H	70	−1	GTG		
tRNA-Ser	11,787	11,854	H	68	0	GCT		
tRNA-Leu	11,855	11,928	H	74	3	TAG		
ND5	11,932	13,752	H	1821	−4		ATA	TAA
ND6	13,749	14,270	L	522	−1		ATG	TAA
tRNA-Glu	14,270	14,339	L	70	3	TTC		
Cytb	14,343	15,479	H	1137	0		ATG	TA-
tRNA-Thr	15,480	15,551	H	72	0	TGT		
Control Region	15,552	16,572	H	1021	0			
tRNA-Pro	16,573	16,643	L	71	0	TGG		
Rep_Region	16,644	16,776	H	133				

**Table 3:** The nucleotide composition of the mitogenomes of *G. manipurensis* and other congeners within the tribe Garrini.

Species Name	Accession No.	Size (bp)	A%	T%	G%	C%	A+T%	AT-Skew	GC-Skew
*Garra manipurensis*	PZ247732	16,776	32.54	28.37	15.08	24.02	60.91	0.069	−0.229
*Garra congoensis*	AP011205	16,761	32.62	27.14	15.15	25.08	59.76	0.092	−0.247
*Garra dengba*	OL826794	16,876	31.83	25.84	15.87	26.45	57.67	0.104	−0.250
*Garra flavatra*	AP011410	16,743	33.34	27.53	14.47	24.66	60.87	0.095	−0.260
*Garra kempi*	KT601639	17,104	32.86	26.04	14.94	26.16	58.90	0.116	−0.273
*Garra lamta*	OR866911	16,854	32.47	26.31	15.32	25.90	58.78	0.105	−0.257
*Garra motuoensis*	OK375462	16,806	32.50	26.37	15.26	25.87	58.87	0.104	−0.258
*Garra orientalis*	AP011202	16,768	32.28	25.66	15.49	26.57	57.94	0.114	−0.264
*Garra poecilura*	AP011411	16,978	32.68	27.21	15.03	25.07	59.90	0.091	−0.250
*Garra qiaojiensis*	KF727438	17,096	33.04	26.30	14.79	25.88	59.34	0.114	−0.273
*Garra rufa*	AP011197	16,763	31.81	26.83	15.98	25.38	58.64	0.085	−0.227
*Garra salweenica*	KF752478	16,960	32.54	25.54	15.21	26.71	58.08	0.120	−0.274
*Garra spilota*	AP011327	16,822	32.67	25.66	15.27	26.40	58.33	0.120	−0.267
*Garra tengchongensis*	OZ352869	16,898	32.71	26.70	15.22	25.36	59.42	0.101	−0.250
*Garra tibetana*	MG999836	16,861	32.31	26.13	15.35	26.20	58.44	0.106	−0.261
*Garra yajiangensis*	OL826795	16,835	32.67	25.98	15.08	26.27	58.65	0.114	−0.271
*Ageneiogarra imberba*	KM255666	16,600	31.48	25.76	16.09	26.67	57.23	0.100	−0.247
*Discocheilus wuluoheensis*	KX840359	16,587	32.12	26.58	15.37	25.94	58.69	0.094	−0.256
*Discocheilus wui*	KX840358	16,589	32.17	26.57	15.29	25.97	58.74	0.095	−0.259
*Tariqilabeo bicornis*	AP013332	16,710	32.81	26.83	15.06	25.30	59.65	0.100	−0.254
*Tariqilabeo burmanicus*	AP013328	16,710	32.79	26.79	15.11	25.31	59.58	0.101	−0.252
*Tariqilabeo latius*	AP012148	16,876	32.95	26.36	14.91	25.79	59.30	0.111	−0.267

## Data Availability

The mitogenome sequence of *G. manipurensis* generated in the present study has been deposited in GenBank (NCBI) under accession number PZ247732 and is accessible at https://www.ncbi.nlm.nih.gov/.

## Supplementary Materials

The following supporting information can be downloaded at: https://www.mdpi.com/article/10.3390/ijms27125555/s1.
