# Supplementary material for: Novel Mitogenome of Garra manipurensis Reveals Gene Rearrangement, Purifying Selection, and Matrilineal Phylogenetic Insights in Garrini (Cypriniformes: Cyprinidae)
Source: Int J Mol Sci. 2026 Jun 19;27(12):5555. doi: 10.3390/ijms27125555 (PMC13299211; doi:10.3390/ijms27125555)
Supplement: Supplementary file 1 [file ijms-27-05555-s001.zip › ijms-4381788-supplementary.pdf]

Supplementary Materials

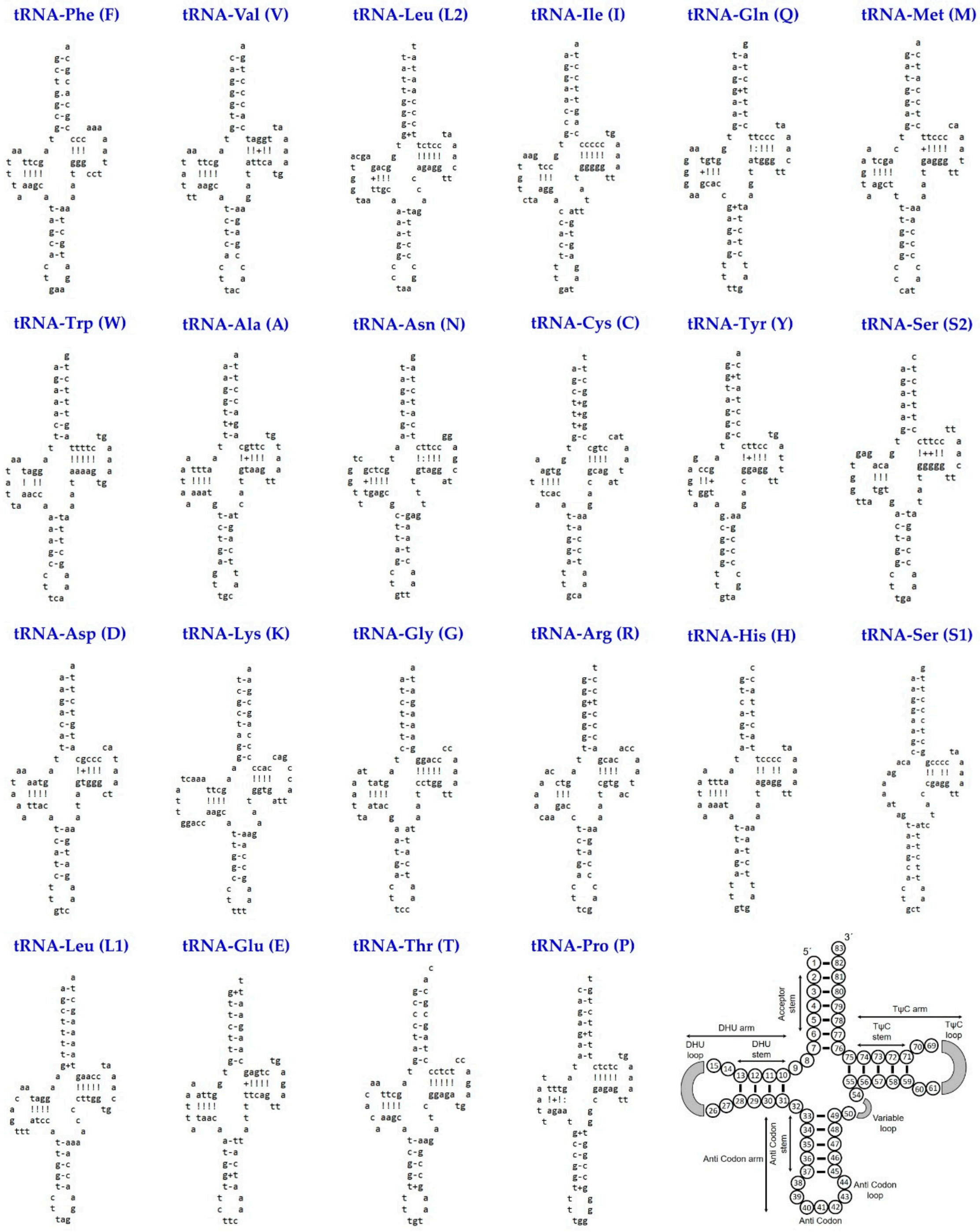

Figure S1. The cloverleaf secondary structures of the 22 tRNAs identified in *G. manipurensis*, showing structural variation. Each tRNA is labeled using the standard three-letter amino acid abbreviation and its corresponding IUPAC–IUB single-letter code.

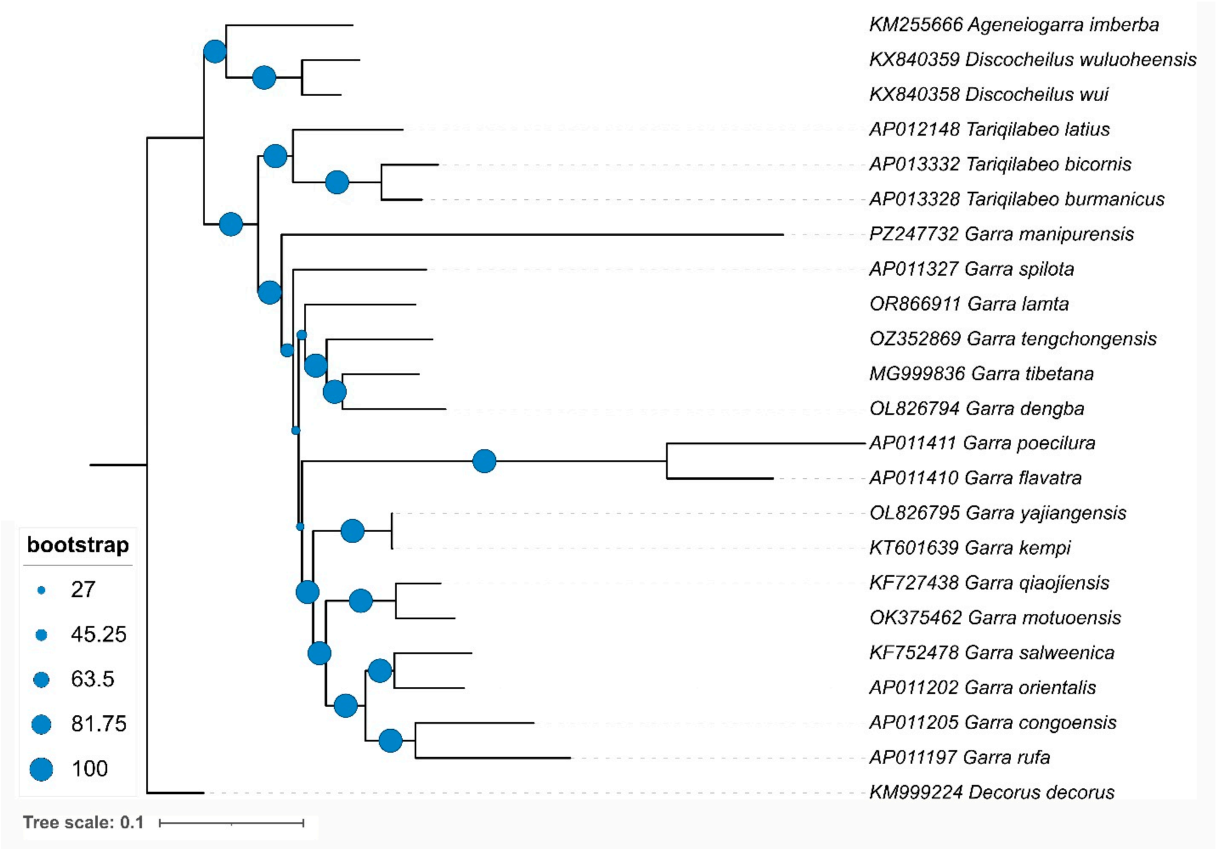

Figure S2. The Maximum Likelihood phylogenetic tree based on the concatenated sequences of 13 PCGs, depicting matrilineal evolutionary relationships among cyprinid species of the tribe Garrini. The bootstrap support values are indicated at each node.

Table S1. The mitogenomic dataset of *G. manipurensis* generated in this study and other Garrini congeners retrieved from GenBank.

No.	Tribe	GenBank Species Name	Valid Species Name	Accession No.	Reference
1	Garrini	*Garra manipurensis*	*Garra manipurensis*	PZ247732	This study
2	Garrini	*Garra congoensis*	*Garra congoensis*	AP011205	Unpublished
3	Garrini	*Garra dengba*	*Garra dengba*	OL826794	[8]
4	Garrini	*Garra flavatra*	*Garra flavatra*	AP011410	[5]
5	Garrini	*Garra kempi*	*Garra kempi*	KT601639	[47]
6	Garrini	*Garra lamta*	*Garra lamta*	OR866911	Unpublished
7	Garrini	*Garra motuoensis*	*Garra motuoensis*	OK375462	[50]
8	Garrini	*Garra orientalis*	*Garra orientalis*	AP011202	[5]
9	Garrini	*Garra poecilura*	*Garra poecilura*	AP011411	Unpublished
10	Garrini	*Garra qiaojiensis*	*Garra qiaojiensis*	KF727438	[30]
11	Garrini	*Garra rufa*	*Garra rufa*	AP011197	[5]
12	Garrini	*Garra salweenica*	*Garra salweenica*	KF752478	Unpublished
13	Garrini	*Garra spilota*	*Garra spilota*	AP011327	[5]
14	Garrini	*Garra tengchongensis*	*Garra tengchongensis*	OZ352869	Unpublished
15	Garrini	*Garra tibetana*	*Garra tibetana*	MG999836	[8]
16	Garrini	*Garra yajiangensis*	*Garra yajiangensis*	OL826795	[8]
17	Garrini	*Garra imberba*	*Ageneiogarra imberba*	KM255666	[36]
18	Garrini	*Discolabeo wuluoheensis*	*Discocheilus wuluoheensis*	KX840359	[48]
19	Garrini	*Discocheilus wui*	*Discocheilus wui*	KX840358	[48]
20	Garrini	*Tariqilabeo bicornis*	*Tariqilabeo bicornis*	AP013332	Unpublished
21	Garrini	*Tariqilabeo burmanicus*	*Tariqilabeo burmanicus*	AP013328	Unpublished
22	Garrini	*Tariqilabeo latius*	*Tariqilabeo latius*	AP012148	[5]
23	Labeonini	*Bangana decora*	*Decorus decorus*	KM999224	[73]

Table S2. The comparative analysis of intergenic nucleotide (IN) regions among the mitogenomes of 16 *Garra* species.

Gene	*G. manipurensis*			*G. congoensis*			*G. dengba*			*G. flavatra*		
	Start	End	IN	Start	End	IN	Start	End	IN	Start	End	IN
tRNA-Phe (F)	1	68	-1	1	69	0	1	69	0	1	69	0
12S rRNA	68	1009	0	70	1021	0	70	1021	-1	70	1019	0
tRNA-Val (V)	1010	1081	18	1022	1093	-2	1021	1092	0	1020	1090	0
16S rRNA	1100	2749	23	1092	2776	0	1093	2780	0	1091	2809	0
tRNA-Leu (L2)	2773	2848	1	2777	2852	1	2781	2856	0	2810	2884	1
ND1	2850	3824	3	2854	3831	0	2857	3831	3	2886	3863	0
tRNA-Ile (I)	3828	3900	-3	3832	3903	-1	3835	3906	-2	3864	3935	-2
tRNA-Gln (Q)	3898	3969	0	3903	3972	0	3905	3975	1	3934	4004	1
tRNA-Met (M)	3970	4039	0	3973	4041	0	3977	4045	0	4006	4074	0
ND2	4040	5084	0	4042	5086	0	4046	5090	0	4075	5119	0
tRNA-Trp (W)	5085	5155	0	5087	5157	2	5091	5161	2	5120	5190	1
tRNA-Ala (A)	5156	5225	0	5160	5228	1	5164	5232	1	5192	5260	1
tRNA-Asn (N)	5226	5299	0	5230	5302	0	5234	5306	0	5262	5334	0
O_L	5300	5327	0	5303	5334	0	5307	5340	0	5335	5366	0
tRNA-Cys (C)	5328	5394	0	5335	5401	1	5341	5406	1	5367	5434	1
tRNA-Tyr (Y)	5395	5462	1	5403	5473	0	5408	5478	1	5436	5504	1
COI	5464	7010	0	5474	7024	0	5480	7030	0	5506	7052	0
tRNA-Ser (S2)	7011	7081	2	7025	7095	3	7031	7101	3	7053	7123	3
tRNA-Asp (D)	7084	7154	8	7099	7168	12	7105	7174	13	7127	7196	7
COII	7163	7853	-1	7181	7871	0	7188	7878	0	7204	7894	0
tRNA-Lys (K)	7853	7931	1	7872	7946	2	7879	7954	1	7895	7971	1
ATP8	7933	8097	-7	7949	8113	-7	7956	8120	-7	7973	8137	-7
ATP6	8091	8774	-1	8107	8789	0	8114	8797	-1	8131	8813	0
COIII	8774	9558	-1	8790	9574	0	8797	9581	0	8814	9598	0
tRNA-Gly (G)	9558	9629	0	9575	9646	0	9582	9653	0	9599	9670	0
ND3	9630	9978	-1	9647	9995	0	9654	10002	0	9671	10019	0
tRNA-Arg (R)	9978	10047	0	9996	10065	0	10003	10072	0	10020	10090	0
ND4L	10048	10344	-7	10066	10362	-7	10073	10369	-7	10091	10387	-7
ND4	10338	11718	-1	10356	11736	0	10363	11743	0	10381	11761	0
tRNA-His (H)	11718	11787	-1	11737	11804	1	11744	11812	0	11762	11830	0
tRNA-Ser (S1)	11787	11854	0	11806	11874	1	11813	11881	1	11831	11899	1
tRNA-Leu (L1)	11855	11928	3	11876	11948	3	11883	11955	3	11901	11973	1
ND5	11932	13752	-4	11952	13775	-4	11959	13782	-4	11975	13792	-5
ND6	13749	14270	-1	13772	14293	1	13779	14300	0	13788	14309	0
tRNA-Glu (E)	14270	14339	3	14295	14362	9	14301	14369	4	14310	14378	5
Cytb	14343	15479	0	14372	15508	4	14374	15514	0	14384	15520	3
tRNA-Thr (T)	15480	15551	0	15513	15585	0	15515	15586	0	15524	15595	0

Control region	15552	16572	0	15586	16443	0	15587	16524	0	15596	16442	0
tRNA-Pro (P)	16573	16643	0	16444	16513	0	16525	16594	0	16443	16512	0
Rep_Region	16644	16776					16595	16876				

**Gene**	***G. kempi***			***G. lamta***			***G. motuoensis***			***G. orientalis***		
	Start	End	IN	Start	End	IN	Start	End	IN	Start	End	IN
tRNA-Phe (F)	1	69	0	1	69	0	1	69	0	1	68	0
12S rRNA	70	1019	0	70	1020	0	70	1022	0	69	1019	0
tRNA-Val (V)	1020	1091	0	1021	1092	0	1023	1094	0	1020	1091	0
16S rRNA	1092	2778	0	1093	2775	0	1095	2780	0	1092	2773	0
tRNA-Leu (L2)	2779	2854	1	2776	2851	1	2781	2856	0	2774	2849	1
ND1	2856	3830	4	2853	3827	4	2857	3831	5	2851	3825	4
tRNA-Ile (I)	3835	3906	-2	3832	3903	-2	3837	3908	-2	3830	3901	-2
tRNA-Gln (Q)	3905	3975	1	3902	3972	1	3907	3977	1	3900	3970	1
tRNA-Met (M)	3977	4045	0	3974	4042	0	3979	4047	0	3972	4040	0
ND2	4046	5090	0	4043	5087	0	4048	5092	0	4041	5085	0
tRNA-Trp (W)	5091	5161	2	5088	5158	2	5093	5163	1	5086	5156	1
tRNA-Ala (A)	5164	5231	1	5161	5229	1	5165	5233	1	5158	5226	1
tRNA-Asn (N)	5233	5305	0	5231	5303	0	5235	5307	0	5228	5300	0
O_L	5306	5338	0	5304	5337	0	5308	5343	-3	5301	5335	0
tRNA-Cys (C)	5339	5405	1	5338	5404	1	5341	5407	1	5336	5402	1
tRNA-Tyr (Y)	5407	5477	1	5406	5476	1	5409	5479	1	5404	5474	1
COI	5479	7029	0	5478	7028	0	5481	7031	0	5476	7026	0
tRNA-Ser (S2)	7030	7100	3	7029	7099	3	7032	7102	3	7027	7097	3
tRNA-Asp (D)	7104	7174	12	7103	7172	13	7106	7184	3	7101	7170	12
COII	7187	7877	0	7186	7876	0	7188	7878	0	7183	7873	0
tRNA-Lys (K)	7878	7953	1	7877	7952	1	7879	7954	1	7874	7949	0
ATP8	7955	8119	-7	7954	8118	-7	7956	8120	-7	7950	8114	-7
ATP6	8113	8796	-1	8112	8794	0	8114	8797	-1	8108	8790	0
COIII	8796	9580	0	8795	9579	0	8797	9581	0	8791	9575	0
tRNA-Gly (G)	9581	9652	0	9580	9651	0	9582	9653	0	9576	9647	0
ND3	9653	10001	0	9652	10000	0	9654	10002	0	9648	9996	0
tRNA-Arg (R)	10002	10071	0	10001	10070	0	10003	10072	0	9997	10066	0
ND4L	10072	10368	-7	10071	10367	-7	10073	10369	-7	10067	10363	-7
ND4	10362	11742	0	10361	11741	0	10363	11743	0	10357	11737	0
tRNA-His (H)	11743	11811	0	11742	11810	0	11744	11812	-1	11738	11806	0
tRNA-Ser (S1)	11812	11879	1	11811	11879	1	11812	11880	2	11807	11874	1
tRNA-Leu (L1)	11881	11953	3	11881	11953	3	11883	11955	3	11876	11948	3
ND5	11957	13780	-4	11957	13777	-4	11959	13782	-4	11952	13775	-4
ND6	13777	14298	0	13774	14295	0	13779	14300	0	13772	14293	0
tRNA-Glu (E)	14299	14367	5	14296	14364	5	14301	14369	5	14294	14362	5
Cytb	14373	15509	4	14370	15510	0	14375	15511	4	14368	15504	4
tRNA-Thr (T)	15514	15585	0	15511	15582	0	15516	15587	0	15509	15580	0
Control region	15586	16499	0	15583	16509	0	15588	16463	0	15581	16427	0

tRNA-Pro (P)	16500	16568	0	16510	16579	0	16464	16533	0	16428	16497	0
Rep_Region	16569	17104		16580	16854		16534	16806				

Gene	*G. poecilura*			*G. qiaojiensis*			*G. rufa*			*G. salweenica*		
	Start	End	IN	Start	End	IN	Start	End	IN	Start	End	IN
tRNA-Phe (F)	1	69	0	1	69	0	1	69	0	1	68	0
12S rRNA	70	1019	0	70	1022	0	70	1022	0	69	1019	0
tRNA-Val (V)	1020	1090	0	1023	1094	0	1023	1094	0	1020	1091	0
16S rRNA	1091	2783	0	1095	2781	0	1095	2776	0	1092	2775	0
tRNA-Leu (L2)	2784	2858	1	2782	2857	1	2777	2852	1	2776	2851	1
ND1	2860	3837	0	2859	3833	4	2854	3828	4	2853	3827	4
tRNA-Ile (I)	3838	3909	-2	3838	3909	-2	3833	3904	-2	3832	3903	-2
tRNA-Gln (Q)	3908	3978	1	3908	3978	1	3903	3973	0	3902	3972	1
tRNA-Met (M)	3980	4048	0	3980	4048	0	3974	4042	0	3974	4042	1
ND2	4049	5093	0	4049	5093	0	4043	5087	0	4044	5088	0
tRNA-Trp (W)	5094	5164	1	5094	5164	1	5088	5158	1	5089	5159	1
tRNA-Ala (A)	5166	5234	1	5166	5234	1	5160	5228	1	5161	5229	1
tRNA-Asn (N)	5236	5308	0	5236	5308	0	5230	5302	0	5231	5303	0
O_L	5309	5340	0	5309	5344	-3	5303	5335	0	5304	5340	-3
tRNA-Cys (C)	5341	5409	1	5342	5408	1	5336	5402	1	5338	5404	1
tRNA-Tyr (Y)	5411	5479	1	5410	5480	1	5404	5474	1	5406	5476	1
COI	5481	7027	0	5482	7032	0	5476	7026	0	5478	7028	0
tRNA-Ser (S2)	7028	7098	3	7033	7103	3	7027	7097	3	7029	7099	3
tRNA-Asp (D)	7102	7171	12	7107	7185	3	7101	7170	12	7103	7172	12
COII	7184	7874	0	7189	7879	0	7183	7873	0	7185	7875	0
tRNA-Lys (K)	7875	7951	1	7880	7955	1	7874	7949	1	7876	7951	1
ATP8	7953	8117	-7	7957	8121	-7	7951	8115	-7	7953	8117	-7
ATP6	8111	8793	0	8115	8798	-1	8109	8791	0	8111	8794	-1
COIII	8794	9578	0	8798	9582	0	8792	9576	0	8794	9578	0
tRNA-Gly (G)	9579	9650	0	9583	9654	0	9577	9648	0	9579	9650	0
ND3	9651	9999	0	9655	10003	0	9649	9997	0	9651	9999	0
tRNA-Arg (R)	10000	10070	0	10004	10073	0	9998	10067	0	10000	10069	0
ND4L	10071	10367	-7	10074	10370	-7	10068	10364	-7	10070	10366	-7
ND4	10361	11741	0	10364	11744	0	10358	11738	0	10360	11740	0
tRNA-His (H)	11742	11810	0	11745	11813	-1	11739	11807	0	11741	11809	-1
tRNA-Ser (S1)	11811	11879	-1	11813	11881	2	11808	11875	1	11809	11876	2
tRNA-Leu (L1)	11879	11951	1	11884	11956	3	11877	11949	3	11879	11951	3
ND5	11953	13770	-5	11960	13783	-4	11953	13776	-4	11955	13781	-4
ND6	13766	14287	0	13780	14301	0	13773	14294	0	13778	14299	0
tRNA-Glu (E)	14288	14356	3	14302	14370	5	14295	14363	8	14300	14368	5
Cytb	14360	15496	3	14376	15512	4	14372	15508	4	14374	15510	4
tRNA-Thr (T)	15500	15570	0	15517	15588	0	15513	15585	0	15515	15587	60
Control region	15571	16680	0	15589	16493	0	15586	16440	0	15648	16475	0
tRNA-Pro (P)	16681	16749	0	16494	16563	14	16441	16510	0	16476	16545	12

Rep_Region				16578	17095					16558	16960	
**Gene**	***G. spilota***			***G. tengchongensis***			***G. tibetana***			***G. yajiangensis***		
	Start	End	IN	Start	End	IN	Start	End	IN	Start	End	IN
tRNA-Phe (F)	1	69	0	1	69	0	1	68	0	1	69	0
12S rRNA	70	1021	0	70	1020	0	69	1019	0	70	1019	0
tRNA-Val (V)	1022	1092	0	1021	1092	0	1020	1091	0	1020	1091	0
16S rRNA	1093	2775	0	1093	2785	-3	1092	2779	0	1092	2778	0
tRNA-Leu (L2)	2776	2851	1	2783	2858	0	2780	2855	1	2779	2854	1
ND1	2853	3827	4	2859	3833	4	2857	3831	4	2856	3830	4
tRNA-Ile (I)	3832	3903	-2	3838	3909	-2	3836	3907	-2	3835	3906	-2
tRNA-Gln (Q)	3902	3972	1	3908	3978	1	3906	3976	1	3905	3975	1
tRNA-Met (M)	3974	4042	0	3980	4048	0	3978	4046	0	3977	4045	0
ND2	4043	5087	0	4049	5093	0	4047	5091	0	4046	5090	0
tRNA-Trp (W)	5088	5158	2	5094	5164	2	5092	5162	2	5091	5161	2
tRNA-Ala (A)	5161	5229	1	5167	5235	2	5165	5233	1	5164	5231	1
tRNA-Asn (N)	5231	5303	-2	5238	5310	0	5235	5307	0	5233	5305	0
O_L	5302	5337	0	5311	5347	-5	5308	5341	0	5306	5338	0
tRNA-Cys (C)	5338	5405	1	5343	5409	1	5342	5409	1	5339	5405	1
tRNA-Tyr (Y)	5407	5477	1	5411	5481	0	5411	5481	1	5407	5474	4
COI	5479	7029	0	5482	7032	0	5483	7033	1	5479	7029	0
tRNA-Ser (S2)	7030	7100	3	7033	7103	3	7035	7103	4	7030	7100	3
tRNA-Asp (D)	7104	7173	13	7107	7176	13	7108	7177	12	7104	7174	12
COII	7187	7877	0	7190	7880	0	7190	7880	0	7187	7877	0
tRNA-Lys (K)	7878	7953	1	7881	7956	1	7881	7956	1	7878	7953	1
ATP8	7955	8119	-7	7958	8122	-7	7958	8122	-7	7955	8119	-7
ATP6	8113	8795	0	8116	8799	-1	8116	8799	-1	8113	8796	-1
COIII	8796	9580	0	8799	9583	0	8799	9583	0	8796	9580	0
tRNA-Gly (G)	9581	9652	0	9584	9655	-1	9584	9654	0	9581	9652	0
ND3	9653	10001	0	9655	10003	0	9655	10003	0	9653	10001	0
tRNA-Arg (R)	10002	10071	0	10004	10073	0	10004	10073	0	10002	10071	0
ND4L	10072	10368	-7	10074	10370	-7	10074	10370	-7	10072	10368	-7
ND4	10362	11742	0	10364	11744	0	10364	11744	0	10362	11742	0
tRNA-His (H)	11743	11811	0	11745	11813	-1	11745	11814	0	11743	11811	0
tRNA-Ser (S1)	11812	11880	0	11813	11881	2	11815	11883	1	11812	11879	1
tRNA-Leu (L1)	11881	11954	4	11884	11956	3	11885	11957	3	11881	11953	3
ND5	11959	13782	-4	11960	13791	-12	11961	13784	-4	11957	13780	-4
ND6	13779	14300	0	13780	14301	0	13781	14302	0	13777	14298	0
tRNA-Glu (E)	14301	14369	5	14302	14370	4	14303	14371	5	14299	14367	5
Cytb	14375	15515	0	14375	15511	4	14377	15517	0	14373	15509	4
tRNA-Thr (T)	15516	15587	0	15516	15588	62	15518	15589	0	15514	15585	0
Control region	15588	16451	0	15651	16517	19	15590	16490	0	15586	16499	0
tRNA-Pro (P)	16452	16521	0	16537	16606	0	16491	16560	0	16500	16568	0
Rep_Region							16561	16861		16569	16835	

Table S3. The comparison of start and stop codons in the 13 PCGs across 16 *Garra* mitogenomes.

Gene	*G. manipurensis*		*G. congoensis*		*G. dengba*		*G. flavatra*		*G. kempi*		*G. lamta*		*G. motuoensis*		*G. orientalis*	
	**Start**	**Stop**	**Start**	**Stop**	**Start**	**Stop**	**Start**	**Stop**	**Start**	**Stop**	**Start**	**Stop**	**Start**	**Stop**	**Start**	**Stop**
ATP6	ATG	TAA	ATG	TA-	ATG	TAA	ATG	TA-	ATG	TAA	ATG	TA-	ATG	TAA	ATG	TA-
ATP8	ATG	TAA	ATG	TAG	ATG	TAG	ATG	TAG	ATG	TAG	ATG	TAA	ATG	TAG	ATG	TAA
COI	GTG	TA-	GTG	TAA	GTG	TAA	GTG	TA-	GTG	TAA	GTG	TAA	GTG	TAA	GTG	TAA
COII	ATG	T--	ATG	T--	ATG	T--	ATG	T--	ATG	T--	ATG	T--	ATG	T--	ATG	T--
COIII	ATG	TA-	ATG	TA-	ATG	TA-	ATG	TA-	ATG	TA-	ATG	TA-	ATG	TA-	ATG	TA-
Cytb	ATG	TA-	ATG	TAA	ATG	T--	ATG	TAG	ATG	TAA	ATG	T--	ATG	TAA	ATG	TAA
ND1	ATG	TAA	ATG	TAA	ATG	TAA	ATG	TAA	ATG	TAA	ATG	TAA	ATG	TAA	ATG	TAA
ND2	ATA	T--	ATG	T--	ATG	T--	ATA	T--	ATG	T--	ATG	T--	ATG	T--	ATG	T--
ND3	ATG	T--	ATG	T--	ATG	T--	ATG	T--	ATG	T--	ATG	T--	ATG	T--	ATG	T--
ND4	ATG	T--	ATG	T--	ATG	T--	ATG	T--	ATG	T--	ATG	T--	ATG	T--	ATG	T--
ND4L	ATG	TAA	ATG	TAA	ATG	TAA	ATG	TAA	ATG	TAA	ATG	TAA	ATG	TAA	ATG	TAA
ND5	ATA	TAA	ATG	TAA	ATA	TAA	ATA	TAA	ATA	TAA	ATA	TAA	ATA	TAA	ATG	TAA
ND6	ATG	TAA	ATG	TAG	ATG	TAG	ATG	AGA	ATG	TAG	ATG	TAG	ATG	TAG	ATG	TAG
**Gene**	*G. poecilura*		*G. qiaojiensis*		*G. rufa*		*G. salweenica*		*G. spilota*		*G. tengchongensis*		*G. tibetana*		*G. yajiangensis*	
	**Start**	**Stop**	**Start**	**Stop**	**Start**	**Stop**	**Start**	**Stop**	**Start**	**Stop**	**Start**	**Stop**	**Start**	**Stop**	**Start**	**Stop**
ATP6	ATG	TA-	ATG	TAA	ATG	TA-	ATG	TAA	ATG	TA-	ATG	TAA	ATG	TAA	ATG	TAA
ATP8	ATG	TAA	ATG	TAG	ATG	TAA	ATG	TAA	ATG	TAA	ATG	TAA	ATG	TAA	ATG	TAG
COI	GTG	TA-	GTG	TAA	GTG	TAA	GTG	TAA	GTG	TAA	GTG	TAA	GTG	TAA	GTG	TAA
COII	ATG	T--	ATG	T--	ATG	T--	ATG	T--	ATG	T--	ATG	T--	ATG	T--	ATG	T--
COIII	ATG	TA-	ATG	TA-	ATG	TA-	ATG	TA-	ATG	TA-	ATG	TA-	ATG	TA-	ATG	TA-
Cytb	ATG	TAA	ATG	TAA	ATG	TAA	ATG	TAA	ATG	T--	ATG	T--	ATG	T--	ATG	TAA
ND1	ATG	TAA	ATG	TAA	ATG	TAA	ATG	TAA	ATG	TAA	ATG	TAG	ATG	TAA	ATG	TAA
ND2	ATA	T--	ATG	T--	ATG	T--	ATG	T--	ATG	T--	ATG	T--	ATG	T--	ATG	T--
ND3	ATG	T--	ATG	T--	ATG	T--	ATG	T--	ATG	T--	ATG	T--	ATG	T--	ATG	T--
ND4	ATG	T--	ATG	T--	ATG	T--	ATG	T--	ATG	T--	ATG	T--	ATG	T--	ATG	T--
ND4L	ATG	TAA	ATG	TAA	ATG	TAA	ATG	TAA	ATG	TAA	ATG	TAA	ATG	TAA	ATG	TAA
ND5	ATA	TAA	ATA	TAA	ATG	TAG	ATG	TAG	ATA	TAA	ATA	TAA	ATA	TAA	ATA	TAA
ND6	ATG	AGA	ATG	TAG	ATG	TAA	ATG	TAG	ATG	TAA	ATG	TAG	ATG	TAA	ATG	TAG

Table S4. The Ka/Ks ratios of the 13 PCGs across different Garrini mitogenomes.

Species	13 PCGs												
	**ND1**	**ND2**	**COI**	**COII**	**ATP8**	**ATP6**	**COIII**	**ND3**	**ND4L**	**ND4**	**ND5**	**ND6**	**Cytb**
G. congoensis	0.065	0.129	0.018	0.054	0.277	0.083	0.048	0.089	0.040	0.098	0.104	0.084	0.064
G. dengba	0.052	0.096	0.017	0.047	0.178	0.072	0.040	0.081	0.031	0.054	0.073	0.059	0.054
G. flavatra	0.129	0.218	0.024	0.028	0.318	0.069	0.052	0.096	0.069	0.170	0.133	0.216	0.078
G. kempi	0.073	0.130	0.019	0.036	0.249	0.061	0.045	0.085	0.036	0.059	0.083	0.066	0.053
G. lamta	0.069	0.077	0.018	0.035	0.201	0.091	0.047	0.048	0.055	0.058	0.087	0.073	0.060
G. motuoensis	0.079	0.109	0.018	0.041	0.172	0.081	0.051	0.096	0.065	0.101	0.095	0.106	0.064
G. orientalis	0.065	0.125	0.021	0.040	0.340	0.086	0.046	0.074	0.031	0.070	0.109	0.062	0.060
G. poecilura	0.117	0.167	0.025	0.052	0.556	0.088	0.050	0.106	0.085	0.170	0.124	0.190	0.068
G. qiaojiensis	0.068	0.126	0.020	0.038	0.143	0.079	0.047	0.119	0.035	0.096	0.089	0.115	0.065
G. rufa	0.056	0.121	0.016	0.054	0.169	0.083	0.045	0.076	0.051	0.070	0.143	0.100	0.060
G. salweenica	0.069	0.149	0.021	0.035	0.389	0.076	0.051	0.091	0.027	0.069	0.106	0.071	0.066
G. spilota	0.057	0.096	0.017	0.043	0.142	0.060	0.044	0.061	0.050	0.059	0.070	0.072	0.050
G. tengchongensis	0.049	0.116	0.017	0.033	0.223	0.077	0.040	0.067	0.050	0.066	0.081	0.056	0.060
G. tibetana	0.056	0.102	0.018	0.041	0.294	0.062	0.044	0.107	0.038	0.071	0.090	0.063	0.075
G. yajiangensis	0.073	0.130	0.019	0.036	0.249	0.061	0.045	0.085	0.038	0.058	0.084	0.064	0.053
A. imberba	0.052	0.099	0.021	0.044	0.190	0.067	0.045	0.100	0.037	0.056	0.088	0.072	0.053
D. wuluoheensis	0.052	0.101	0.022	0.045	0.207	0.074	0.046	0.090	0.044	0.062	0.089	0.063	0.060
D. wui	0.055	0.107	0.019	0.048	0.273	0.083	0.050	0.078	0.034	0.064	0.090	0.067	0.058
T. bicornis	0.056	0.137	0.023	0.042	0.374	0.079	0.049	0.090	0.039	0.102	0.086	0.078	0.058
T. burmanicus	0.059	0.121	0.024	0.040	0.373	0.081	0.052	0.075	0.044	0.091	0.084	0.079	0.057
T. latius	0.055	0.127	0.019	0.039	0.197	0.079	0.050	0.082	0.043	0.065	0.079	0.056	0.054
Average	0.069	0.126	0.020	0.041	0.261	0.075	0.047	0.087	0.046	0.085	0.097	0.094	0.061
STDEV	0.023	0.033	0.002	0.007	0.100	0.009	0.004	0.018	0.015	0.037	0.021	0.053	0.007

Table S5. The RSCU values of the 13 PCGs in 16 *Garra* mitogenomes.

G. manipurensis											
Codon	Count	RSCU	Codon	Count	RSCU	Codon	Count	RSCU	Codon	Count	RSCU
UUU(F)	126	1.30	UCU(S)	67	1.28	UAU(Y)	140	1.26	UGU(C)	38	0.82
UUC(F)	68	0.70	UCC(S)	48	0.92	UAC(Y)	83	0.74	UGC(C)	55	1.18
UUA(L)	137	1.69	UCA(S)	83	1.59	UAA(*)	98	1.37	UGA(*)	54	0.76
UUG(L)	62	0.77	UCG(S)	13	0.25	UAG(*)	62	0.87	UGG(W)	51	1.00
CUU(L)	91	1.13	CCU(P)	87	1.23	CAU(H)	64	1.01	CGU(R)	22	0.73
CUC(L)	45	0.56	CCC(P)	84	1.18	CAC(H)	63	0.99	CGC(R)	22	0.73
CUA(L)	108	1.34	CCA(P)	83	1.17	CAA(Q)	79	1.37	CGA(R)	21	0.70
CUG(L)	42	0.52	CCG(P)	30	0.42	CAG(Q)	36	0.63	CGG(R)	26	0.87
AUU(I)	163	1.59	ACU(T)	109	1.35	AAU(N)	132	1.16	AGU(S)	39	0.75
AUC(I)	54	0.53	ACC(T)	91	1.13	AAC(N)	96	0.84	AGC(S)	63	1.21
AUA(I)	90	0.88	ACA(T)	100	1.24	AAA(K)	85	1.55	AGA(R)	33	1.10
AUG(M)	60	1.00	ACG(T)	22	0.27	AAG(K)	25	0.45	AGG(R)	56	1.87
GUU(V)	21	0.92	GCU(A)	34	0.96	GAU(D)	32	0.96	GGU(G)	22	0.76
GUC(V)	15	0.66	GCC(A)	57	1.61	GAC(D)	35	1.04	GGC(G)	28	0.97
GUA(V)	33	1.45	GCA(A)	40	1.13	GAA(E)	51	1.32	GGA(G)	31	1.07
GUG(V)	22	0.97	GCG(A)	11	0.31	GAG(E)	26	0.68	GGG(G)	35	1.21
G. congoensis											
Codon	Count	RSCU	Codon	Count	RSCU	Codon	Count	RSCU	Codon	Count	RSCU
UUU(F)	88	1.10	UCU(S)	88	1.45	UAU(Y)	126	1.24	UGU(C)	34	0.79
UUC(F)	72	0.90	UCC(S)	58	0.95	UAC(Y)	78	0.76	UGC(C)	52	1.21
UUA(L)	141	1.77	UCA(S)	76	1.25	UAA(*)	99	1.28	UGA(*)	50	0.65
UUG(L)	59	0.74	UCG(S)	25	0.41	UAG(*)	83	1.07	UGG(W)	44	1.00
CUU(L)	75	0.94	CCU(P)	99	1.34	CAU(H)	75	0.97	CGU(R)	22	0.63
CUC(L)	51	0.64	CCC(P)	90	1.22	CAC(H)	80	1.03	CGC(R)	25	0.72
CUA(L)	96	1.21	CCA(P)	85	1.15	CAA(Q)	91	1.26	CGA(R)	26	0.75
CUG(L)	55	0.69	CCG(P)	22	0.30	CAG(Q)	53	0.74	CGG(R)	25	0.72
AUU(I)	140	1.44	ACU(T)	106	1.37	AAU(N)	131	1.23	AGU(S)	45	0.74
AUC(I)	68	0.70	ACC(T)	77	0.99	AAC(N)	82	0.77	AGC(S)	73	1.20

AUA(I)	83	0.86	ACA(T)	98	1.26	AAA(K)	83	1.48	AGA(R)	52	1.49
AUG(M)	65	1.00	ACG(T)	29	0.37	AAG(K)	29	0.52	AGG(R)	59	1.69
GUU(V)	21	1.09	GCU(A)	20	0.68	GAU(D)	41	1.09	GGU(G)	21	0.80
GUC(V)	15	0.78	GCC(A)	50	1.69	GAC(D)	34	0.91	GGC(G)	24	0.91
GUA(V)	25	1.30	GCA(A)	44	1.49	GAA(E)	40	1.25	GGA(G)	28	1.07
GUG(V)	16	0.83	GCG(A)	4	0.14	GAG(E)	24	0.75	GGG(G)	32	1.22
G. dengba											
Codon	Count	RSCU	Codon	Count	RSCU	Codon	Count	RSCU	Codon	Count	RSCU
UUU(F)	95	1.04	UCU(S)	56	1.09	UAU(Y)	103	1.07	UGU(C)	35	1.01
UUC(F)	87	0.96	UCC(S)	71	1.39	UAC(Y)	89	0.93	UGC(C)	34	0.99
UUA(L)	130	1.51	UCA(S)	79	1.54	UAA(*)	73	1.18	UGA(*)	60	0.97
UUG(L)	51	0.59	UCG(S)	18	0.35	UAG(*)	53	0.85	UGG(W)	37	1.00
CUU(L)	77	0.90	CCU(P)	59	0.83	CAU(H)	60	0.92	CGU(R)	16	0.59
CUC(L)	53	0.62	CCC(P)	94	1.31	CAC(H)	70	1.08	CGC(R)	20	0.74
CUA(L)	158	1.84	CCA(P)	100	1.40	CAA(Q)	81	1.29	CGA(R)	40	1.48
CUG(L)	46	0.54	CCG(P)	33	0.46	CAG(Q)	45	0.71	CGG(R)	23	0.85
AUU(I)	125	1.24	ACU(T)	71	0.98	AAU(N)	98	1.09	AGU(S)	38	0.74
AUC(I)	88	0.87	ACC(T)	99	1.37	AAC(N)	81	0.91	AGC(S)	45	0.88
AUA(I)	90	0.89	ACA(T)	100	1.38	AAA(K)	84	1.56	AGA(R)	36	1.33
AUG(M)	38	1.00	ACG(T)	20	0.28	AAG(K)	24	0.44	AGG(R)	27	1.00
GUU(V)	33	1.14	GCU(A)	43	0.72	GAU(D)	49	1.01	GGU(G)	18	0.46
GUC(V)	25	0.86	GCC(A)	108	1.82	GAC(D)	48	0.99	GGC(G)	39	0.99
GUA(V)	39	1.34	GCA(A)	70	1.18	GAA(E)	55	1.34	GGA(G)	47	1.19
GUG(V)	19	0.66	GCG(A)	17	0.29	GAG(E)	27	0.66	GGG(G)	54	1.37
G. flavatra											
Codon	Count	RSCU	Codon	Count	RSCU	Codon	Count	RSCU	Codon	Count	RSCU
UUU(F)	131	1.37	UCU(S)	56	1.08	UAU(Y)	128	1.28	UGU(C)	48	0.93
UUC(F)	60	0.63	UCC(S)	45	0.87	UAC(Y)	72	0.72	UGC(C)	55	1.07
UUA(L)	106	1.39	UCA(S)	54	1.04	UAA(*)	48	1.01	UGA(*)	81	1.71
UUG(L)	24	0.31	UCG(S)	12	0.23	UAG(*)	13	0.27	UGG(W)	51	1.00
CUU(L)	84	1.10	CCU(P)	94	1.38	CAU(H)	68	1.05	CGU(R)	22	0.61
CUC(L)	55	0.72	CCC(P)	73	1.07	CAC(H)	62	0.95	CGC(R)	38	1.06

CUA(L)	151	1.98	CCA(P)	94	1.38	CAA(Q)	79	1.86	CGA(R)	29	0.81
CUG(L)	38	0.50	CCG(P)	11	0.16	CAG(Q)	6	0.14	CGG(R)	19	0.53
AUU(I)	195	1.51	ACU(T)	113	1.38	AAU(N)	141	1.18	AGU(S)	58	1.12
AUC(I)	78	0.60	ACC(T)	96	1.17	AAC(N)	99	0.83	AGC(S)	86	1.66
AUA(I)	114	0.88	ACA(T)	107	1.30	AAA(K)	99	1.71	AGA(R)	46	1.28
AUG(M)	40	1.00	ACG(T)	12	0.15	AAG(K)	17	0.29	AGG(R)	61	1.70
GUU(V)	38	1.43	GCU(A)	36	0.81	GAU(D)	24	1.04	GGU(G)	12	0.35
GUC(V)	13	0.49	GCC(A)	75	1.69	GAC(D)	22	0.96	GGC(G)	42	1.21
GUA(V)	43	1.62	GCA(A)	61	1.38	GAA(E)	54	1.74	GGA(G)	61	1.76
GUG(V)	12	0.45	GCG(A)	5	0.11	GAG(E)	8	0.26	GGG(G)	24	0.69
G. kempi											
Codon	Count	RSCU	Codon	Count	RSCU	Codon	Count	RSCU	Codon	Count	RSCU
UUU(F)	87	0.94	UCU(S)	59	1.10	UAU(Y)	103	1.14	UGU(C)	28	0.88
UUC(F)	98	1.06	UCC(S)	73	1.36	UAC(Y)	77	0.86	UGC(C)	36	1.13
UUA(L)	125	1.46	UCA(S)	92	1.72	UAA(*)	72	1.10	UGA(*)	66	1.01
UUG(L)	56	0.65	UCG(S)	17	0.32	UAG(*)	59	0.90	UGG(W)	27	1.00
CUU(L)	72	0.84	CCU(P)	63	0.95	CAU(H)	56	0.78	CGU(R)	19	0.72
CUC(L)	52	0.61	CCC(P)	91	1.37	CAC(H)	87	1.22	CGC(R)	20	0.76
CUA(L)	171	1.99	CCA(P)	89	1.34	CAA(Q)	92	1.34	CGA(R)	36	1.37
CUG(L)	39	0.45	CCG(P)	23	0.35	CAG(Q)	45	0.66	CGG(R)	19	0.72
AUU(I)	139	1.30	ACU(T)	76	1.02	AAU(N)	97	1.03	AGU(S)	33	0.62
AUC(I)	86	0.80	ACC(T)	100	1.34	AAC(N)	91	0.97	AGC(S)	47	0.88
AUA(I)	97	0.90	ACA(T)	103	1.38	AAA(K)	86	1.52	AGA(R)	35	1.33
AUG(M)	36	1.00	ACG(T)	19	0.26	AAG(K)	27	0.48	AGG(R)	29	1.10
GUU(V)	37	1.35	GCU(A)	44	0.82	GAU(D)	49	1.05	GGU(G)	15	0.40
GUC(V)	14	0.51	GCC(A)	93	1.73	GAC(D)	44	0.95	GGC(G)	32	0.86
GUA(V)	44	1.60	GCA(A)	74	1.38	GAA(E)	54	1.27	GGA(G)	69	1.85
GUG(V)	15	0.55	GCG(A)	4	0.07	GAG(E)	31	0.73	GGG(G)	33	0.89
G. lamta											
Codon	Count	RSCU	Codon	Count	RSCU	Codon	Count	RSCU	Codon	Count	RSCU
UUU(F)	86	1.15	UCU(S)	83	1.33	UAU(Y)	127	1.22	UGU(C)	34	0.89
UUC(F)	63	0.85	UCC(S)	69	1.10	UAC(Y)	82	0.78	UGC(C)	42	1.11

UUA(L)	121	1.54	UCA(S)	70	1.12	UAA(*)	97	1.24	UGA(*)	57	0.73
UUG(L)	70	0.89	UCG(S)	23	0.37	UAG(*)	81	1.03	UGG(W)	39	1.00
CUU(L)	77	0.98	CCU(P)	102	1.27	CAU(H)	74	0.96	CGU(R)	23	0.64
CUC(L)	44	0.56	CCC(P)	90	1.12	CAC(H)	80	1.04	CGC(R)	29	0.81
CUA(L)	105	1.33	CCA(P)	101	1.25	CAA(Q)	91	1.27	CGA(R)	24	0.67
CUG(L)	55	0.70	CCG(P)	29	0.36	CAG(Q)	52	0.73	CGG(R)	26	0.73
AUU(I)	125	1.39	ACU(T)	100	1.22	AAU(N)	117	1.13	AGU(S)	51	0.82
AUC(I)	68	0.76	ACC(T)	95	1.16	AAC(N)	90	0.87	AGC(S)	79	1.26
AUA(I)	77	0.86	ACA(T)	98	1.20	AAA(K)	78	1.47	AGA(R)	45	1.26
AUG(M)	55	1.00	ACG(T)	35	0.43	AAG(K)	28	0.53	AGG(R)	68	1.90
GUU(V)	17	0.92	GCU(A)	29	0.91	GAU(D)	33	0.94	GGU(G)	21	0.84
GUC(V)	18	0.97	GCC(A)	51	1.59	GAC(D)	37	1.06	GGC(G)	25	1.00
GUA(V)	28	1.51	GCA(A)	42	1.31	GAA(E)	42	1.12	GGA(G)	30	1.20
GUG(V)	11	0.59	GCG(A)	6	0.19	GAG(E)	33	0.88	GGG(G)	24	0.96
G. motuoensis											
Codon	Count	RSCU	Codon	Count	RSCU	Codon	Count	RSCU	Codon	Count	RSCU
UUU(F)	90	0.95	UCU(S)	66	1.24	UAU(Y)	106	1.15	UGU(C)	31	0.95
UUC(F)	99	1.05	UCC(S)	66	1.24	UAC(Y)	79	0.85	UGC(C)	34	1.05
UUA(L)	132	1.51	UCA(S)	77	1.44	UAA(*)	76	1.10	UGA(*)	71	1.02
UUG(L)	50	0.57	UCG(S)	24	0.45	UAG(*)	61	0.88	UGG(W)	25	1.00
CUU(L)	85	0.97	CCU(P)	60	0.91	CAU(H)	60	0.86	CGU(R)	20	0.76
CUC(L)	51	0.58	CCC(P)	84	1.28	CAC(H)	80	1.14	CGC(R)	23	0.88
CUA(L)	164	1.87	CCA(P)	92	1.40	CAA(Q)	90	1.42	CGA(R)	30	1.15
CUG(L)	43	0.49	CCG(P)	27	0.41	CAG(Q)	37	0.58	CGG(R)	22	0.84
AUU(I)	162	1.48	ACU(T)	66	0.93	AAU(N)	100	1.10	AGU(S)	29	0.54
AUC(I)	70	0.64	ACC(T)	102	1.43	AAC(N)	81	0.90	AGC(S)	58	1.09
AUA(I)	97	0.88	ACA(T)	103	1.45	AAA(K)	78	1.43	AGA(R)	33	1.26
AUG(M)	35	1.00	ACG(T)	14	0.20	AAG(K)	31	0.57	AGG(R)	29	1.11
GUU(V)	31	1.10	GCU(A)	38	0.70	GAU(D)	51	1.10	GGU(G)	26	0.68
GUC(V)	23	0.81	GCC(A)	92	1.70	GAC(D)	42	0.90	GGC(G)	20	0.53
GUA(V)	43	1.52	GCA(A)	78	1.44	GAA(E)	50	1.19	GGA(G)	70	1.84
GUG(V)	16	0.57	GCG(A)	9	0.17	GAG(E)	34	0.81	GGG(G)	36	0.95

G. orientalis											
Codon	Count	RSCU	Codon	Count	RSCU	Codon	Count	RSCU	Codon	Count	RSCU
UUU(F)	83	1.15	UCU(S)	80	1.32	UAU(Y)	118	1.16	UGU(C)	35	0.92
UUC(F)	61	0.85	UCC(S)	61	1.01	UAC(Y)	86	0.84	UGC(C)	41	1.08
UUA(L)	118	1.48	UCA(S)	77	1.27	UAA(*)	93	1.26	UGA(*)	53	0.72
UUG(L)	64	0.81	UCG(S)	20	0.33	UAG(*)	76	1.03	UGG(W)	42	1.00
CUU(L)	81	1.02	CCU(P)	103	1.23	CAU(H)	70	0.94	CGU(R)	27	0.72
CUC(L)	53	0.67	CCC(P)	100	1.19	CAC(H)	79	1.06	CGC(R)	32	0.86
CUA(L)	115	1.45	CCA(P)	100	1.19	CAA(Q)	91	1.27	CGA(R)	30	0.80
CUG(L)	46	0.58	CCG(P)	33	0.39	CAG(Q)	52	0.73	CGG(R)	32	0.86
AUU(I)	115	1.38	ACU(T)	100	1.24	AAU(N)	118	1.15	AGU(S)	48	0.79
AUC(I)	59	0.71	ACC(T)	96	1.19	AAC(N)	87	0.85	AGC(S)	77	1.27
AUA(I)	76	0.91	ACA(T)	96	1.19	AAA(K)	78	1.46	AGA(R)	46	1.23
AUG(M)	58	1.00	ACG(T)	30	0.37	AAG(K)	29	0.54	AGG(R)	57	1.53
GUU(V)	19	0.97	GCU(A)	27	0.73	GAU(D)	31	0.84	GGU(G)	19	0.70
GUC(V)	21	1.08	GCC(A)	61	1.66	GAC(D)	43	1.16	GGC(G)	21	0.77
GUA(V)	25	1.28	GCA(A)	49	1.33	GAA(E)	40	1.13	GGA(G)	27	0.99
GUG(V)	13	0.67	GCG(A)	10	0.27	GAG(E)	31	0.87	GGG(G)	42	1.54
G. poecilura											
Codon	Count	RSCU	Codon	Count	RSCU	Codon	Count	RSCU	Codon	Count	RSCU
UUU(F)	112	1.30	UCU(S)	61	1.14	UAU(Y)	124	1.32	UGU(C)	48	0.87
UUC(F)	60	0.70	UCC(S)	50	0.93	UAC(Y)	64	0.68	UGC(C)	62	1.13
UUA(L)	102	1.27	UCA(S)	63	1.18	UAA(*)	49	1.08	UGA(*)	75	1.65
UUG(L)	22	0.27	UCG(S)	11	0.21	UAG(*)	12	0.26	UGG(W)	57	1.00
CUU(L)	91	1.13	CCU(P)	84	1.34	CAU(H)	53	0.75	CGU(R)	22	0.64
CUC(L)	77	0.96	CCC(P)	72	1.15	CAC(H)	89	1.25	CGC(R)	32	0.93
CUA(L)	140	1.74	CCA(P)	80	1.27	CAA(Q)	69	1.66	CGA(R)	36	1.04
CUG(L)	51	0.63	CCG(P)	15	0.24	CAG(Q)	14	0.34	CGG(R)	21	0.61
AUU(I)	168	1.47	ACU(T)	122	1.47	AAU(N)	140	1.20	AGU(S)	48	0.90
AUC(I)	72	0.63	ACC(T)	93	1.12	AAC(N)	94	0.80	AGC(S)	88	1.64
AUA(I)	104	0.91	ACA(T)	102	1.23	AAA(K)	104	1.76	AGA(R)	40	1.16
AUG(M)	38	1.00	ACG(T)	15	0.18	AAG(K)	14	0.24	AGG(R)	56	1.62

GUU(V)	30	1.09	GCU(A)	54	1.07	GAU(D)	27	0.98	GGU(G)	28	0.73
GUC(V)	23	0.84	GCC(A)	74	1.47	GAC(D)	28	1.02	GGC(G)	36	0.94
GUA(V)	42	1.53	GCA(A)	68	1.35	GAA(E)	45	1.45	GGA(G)	55	1.43
GUG(V)	15	0.55	GCG(A)	6	0.12	GAG(E)	17	0.55	GGG(G)	35	0.91
G. qiaojiensis											
Codon	Count	RSCU	Codon	Count	RSCU	Codon	Count	RSCU	Codon	Count	RSCU
UUU(F)	87	0.97	UCU(S)	65	1.23	UAU(Y)	104	1.11	UGU(C)	28	0.90
UUC(F)	93	1.03	UCC(S)	72	1.37	UAC(Y)	83	0.89	UGC(C)	34	1.10
UUA(L)	138	1.55	UCA(S)	78	1.48	UAA(*)	73	1.10	UGA(*)	67	1.01
UUG(L)	53	0.60	UCG(S)	18	0.34	UAG(*)	60	0.90	UGG(W)	25	1.00
CUU(L)	84	0.95	CCU(P)	62	0.94	CAU(H)	62	0.86	CGU(R)	20	0.76
CUC(L)	58	0.65	CCC(P)	84	1.27	CAC(H)	83	1.14	CGC(R)	19	0.72
CUA(L)	166	1.87	CCA(P)	92	1.39	CAA(Q)	94	1.42	CGA(R)	34	1.29
CUG(L)	34	0.38	CCG(P)	27	0.41	CAG(Q)	38	0.58	CGG(R)	19	0.72
AUU(I)	155	1.42	ACU(T)	67	0.96	AAU(N)	102	1.07	AGU(S)	31	0.59
AUC(I)	71	0.65	ACC(T)	96	1.38	AAC(N)	88	0.93	AGC(S)	52	0.99
AUA(I)	102	0.93	ACA(T)	99	1.42	AAA(K)	81	1.50	AGA(R)	34	1.29
AUG(M)	34	1.00	ACG(T)	17	0.24	AAG(K)	27	0.50	AGG(R)	32	1.22
GUU(V)	34	1.18	GCU(A)	38	0.70	GAU(D)	49	1.08	GGU(G)	18	0.49
GUC(V)	23	0.80	GCC(A)	94	1.72	GAC(D)	42	0.92	GGC(G)	25	0.68
GUA(V)	43	1.50	GCA(A)	77	1.41	GAA(E)	58	1.29	GGA(G)	74	2.03
GUG(V)	15	0.52	GCG(A)	9	0.17	GAG(E)	32	0.71	GGG(G)	29	0.79
G. rufa											
Codon	Count	RSCU	Codon	Count	RSCU	Codon	Count	RSCU	Codon	Count	RSCU
UUU(F)	100	1.18	UCU(S)	79	1.34	UAU(Y)	114	1.17	UGU(C)	43	0.93
UUC(F)	69	0.82	UCC(S)	59	1.00	UAC(Y)	81	0.83	UGC(C)	49	1.07
UUA(L)	122	1.53	UCA(S)	73	1.23	UAA(*)	93	1.23	UGA(*)	58	0.77
UUG(L)	68	0.85	UCG(S)	23	0.39	UAG(*)	75	1.00	UGG(W)	53	1.00
CUU(L)	72	0.90	CCU(P)	96	1.25	CAU(H)	81	1.07	CGU(R)	21	0.61
CUC(L)	48	0.60	CCC(P)	92	1.20	CAC(H)	71	0.93	CGC(R)	33	0.96
CUA(L)	112	1.41	CCA(P)	88	1.15	CAA(Q)	94	1.31	CGA(R)	21	0.61
CUG(L)	56	0.70	CCG(P)	30	0.39	CAG(Q)	50	0.69	CGG(R)	23	0.67

AUU(I)	123	1.39	ACU(T)	96	1.23	AAU(N)	119	1.20	AGU(S)	51	0.86
AUC(I)	68	0.77	ACC(T)	88	1.13	AAC(N)	80	0.80	AGC(S)	70	1.18
AUA(I)	74	0.84	ACA(T)	94	1.21	AAA(K)	75	1.44	AGA(R)	50	1.45
AUG(M)	59	1.00	ACG(T)	34	0.44	AAG(K)	29	0.56	AGG(R)	59	1.71
GUU(V)	28	1.30	GCU(A)	38	1.07	GAU(D)	37	1.06	GGU(G)	23	0.79
GUC(V)	13	0.60	GCC(A)	52	1.46	GAC(D)	33	0.94	GGC(G)	26	0.89
GUA(V)	29	1.35	GCA(A)	41	1.15	GAA(E)	38	1.09	GGA(G)	32	1.09
GUG(V)	16	0.74	GCG(A)	11	0.31	GAG(E)	32	0.91	GGG(G)	36	1.23
G. salweenica											
Codon	Count	RSCU	Codon	Count	RSCU	Codon	Count	RSCU	Codon	Count	RSCU
UUU(F)	83	0.95	UCU(S)	62	1.21	UAU(Y)	92	1.04	UGU(C)	33	1.05
UUC(F)	92	1.05	UCC(S)	70	1.36	UAC(Y)	85	0.96	UGC(C)	30	0.95
UUA(L)	130	1.45	UCA(S)	83	1.62	UAA(*)	77	1.18	UGA(*)	66	1.01
UUG(L)	48	0.54	UCG(S)	18	0.35	UAG(*)	53	0.81	UGG(W)	30	1.00
CUU(L)	80	0.89	CCU(P)	59	0.90	CAU(H)	69	0.95	CGU(R)	20	0.74
CUC(L)	61	0.68	CCC(P)	91	1.39	CAC(H)	77	1.05	CGC(R)	26	0.96
CUA(L)	173	1.93	CCA(P)	88	1.35	CAA(Q)	90	1.36	CGA(R)	37	1.37
CUG(L)	45	0.50	CCG(P)	23	0.35	CAG(Q)	42	0.64	CGG(R)	16	0.59
AUU(I)	124	1.25	ACU(T)	69	0.91	AAU(N)	99	1.06	AGU(S)	25	0.49
AUC(I)	85	0.86	ACC(T)	114	1.50	AAC(N)	88	0.94	AGC(S)	50	0.97
AUA(I)	89	0.90	ACA(T)	97	1.28	AAA(K)	87	1.46	AGA(R)	35	1.30
AUG(M)	37	1.00	ACG(T)	23	0.30	AAG(K)	32	0.54	AGG(R)	28	1.04
GUU(V)	33	1.06	GCU(A)	45	0.83	GAU(D)	47	1.04	GGU(G)	23	0.57
GUC(V)	26	0.83	GCC(A)	88	1.61	GAC(D)	43	0.96	GGC(G)	34	0.84
GUA(V)	46	1.47	GCA(A)	78	1.43	GAA(E)	50	1.28	GGA(G)	69	1.71
GUG(V)	20	0.64	GCG(A)	7	0.13	GAG(E)	28	0.72	GGG(G)	35	0.87
G. spilota											
Codon	Count	RSCU	Codon	Count	RSCU	Codon	Count	RSCU	Codon	Count	RSCU
UUU(F)	78	1.05	UCU(S)	93	1.50	UAU(Y)	113	1.18	UGU(C)	28	0.79
UUC(F)	70	0.95	UCC(S)	58	0.94	UAC(Y)	79	0.82	UGC(C)	43	1.21
UUA(L)	98	1.28	UCA(S)	75	1.21	UAA(*)	96	1.26	UGA(*)	54	0.71
UUG(L)	60	0.78	UCG(S)	28	0.45	UAG(*)	79	1.03	UGG(W)	45	1.00

CUU(L)	69	0.90	CCU(P)	102	1.26	CAU(H)	82	0.99	CGU(R)	29	0.74
CUC(L)	54	0.71	CCC(P)	88	1.08	CAC(H)	83	1.01	CGC(R)	32	0.82
CUA(L)	133	1.74	CCA(P)	103	1.27	CAA(Q)	89	1.31	CGA(R)	33	0.84
CUG(L)	45	0.59	CCG(P)	32	0.39	CAG(Q)	47	0.69	CGG(R)	29	0.74
AUU(I)	113	1.27	ACU(T)	107	1.28	AAU(N)	121	1.13	AGU(S)	47	0.76
AUC(I)	81	0.91	ACC(T)	90	1.08	AAC(N)	94	0.87	AGC(S)	70	1.13
AUA(I)	73	0.82	ACA(T)	100	1.20	AAA(K)	81	1.47	AGA(R)	46	1.17
AUG(M)	56	1.00	ACG(T)	37	0.44	AAG(K)	29	0.53	AGG(R)	66	1.69
GUU(V)	16	0.88	GCU(A)	31	0.95	GAU(D)	35	0.97	GGU(G)	17	0.72
GUC(V)	18	0.99	GCC(A)	51	1.56	GAC(D)	37	1.03	GGC(G)	23	0.97
GUA(V)	27	1.48	GCA(A)	43	1.31	GAA(E)	39	1.05	GGA(G)	35	1.47
GUG(V)	12	0.66	GCG(A)	6	0.18	GAG(E)	35	0.95	GGG(G)	20	0.84
G. tengchongensis											
Codon	Count	RSCU	Codon	Count	RSCU	Codon	Count	RSCU	Codon	Count	RSCU
UUU(F)	108	1.12	UCU(S)	60	1.21	UAU(Y)	104	1.16	UGU(C)	34	1.03
UUC(F)	85	0.88	UCC(S)	69	1.39	UAC(Y)	75	0.84	UGC(C)	32	0.97
UUA(L)	156	1.79	UCA(S)	76	1.54	UAA(*)	82	1.17	UGA(*)	65	0.92
UUG(L)	46	0.53	UCG(S)	17	0.34	UAG(*)	64	0.91	UGG(W)	23	1.00
CUU(L)	84	0.97	CCU(P)	54	0.83	CAU(H)	66	0.90	CGU(R)	19	0.70
CUC(L)	45	0.52	CCC(P)	86	1.33	CAC(H)	80	1.10	CGC(R)	20	0.73
CUA(L)	155	1.78	CCA(P)	94	1.45	CAA(Q)	90	1.37	CGA(R)	35	1.28
CUG(L)	36	0.41	CCG(P)	25	0.39	CAG(Q)	41	0.63	CGG(R)	22	0.80
AUU(I)	140	1.27	ACU(T)	65	0.92	AAU(N)	118	1.22	AGU(S)	32	0.65
AUC(I)	85	0.77	ACC(T)	94	1.33	AAC(N)	75	0.78	AGC(S)	43	0.87
AUA(I)	105	0.95	ACA(T)	98	1.39	AAA(K)	83	1.51	AGA(R)	36	1.32
AUG(M)	31	1.00	ACG(T)	25	0.35	AAG(K)	27	0.49	AGG(R)	32	1.17
GUU(V)	36	1.23	GCU(A)	49	0.88	GAU(D)	43	0.96	GGU(G)	27	0.72
GUC(V)	19	0.65	GCC(A)	90	1.62	GAC(D)	47	1.04	GGC(G)	28	0.74
GUA(V)	48	1.64	GCA(A)	73	1.32	GAA(E)	53	1.23	GGA(G)	62	1.64
GUG(V)	14	0.48	GCG(A)	10	0.18	GAG(E)	33	0.77	GGG(G)	34	0.90
G. tibetana											
Codon	Count	RSCU	Codon	Count	RSCU	Codon	Count	RSCU	Codon	Count	RSCU

UUU(F)	97	1.08	UCU(S)	58	1.11	UAU(Y)	102	1.11	UGU(C)	39	1.07
UUC(F)	82	0.92	UCC(S)	72	1.38	UAC(Y)	82	0.89	UGC(C)	34	0.93
UUA(L)	134	1.54	UCA(S)	81	1.55	UAA(*)	79	1.23	UGA(*)	59	0.92
UUG(L)	51	0.59	UCG(S)	18	0.34	UAG(*)	55	0.85	UGG(W)	34	1.00
CUU(L)	69	0.79	CCU(P)	66	0.97	CAU(H)	64	0.88	CGU(R)	15	0.63
CUC(L)	58	0.67	CCC(P)	86	1.26	CAC(H)	81	1.12	CGC(R)	16	0.67
CUA(L)	173	1.99	CCA(P)	96	1.41	CAA(Q)	82	1.24	CGA(R)	36	1.51
CUG(L)	37	0.43	CCG(P)	24	0.35	CAG(Q)	50	0.76	CGG(R)	17	0.71
AUU(I)	128	1.24	ACU(T)	72	0.96	AAU(N)	97	1.08	AGU(S)	39	0.75
AUC(I)	80	0.77	ACC(T)	109	1.45	AAC(N)	83	0.92	AGC(S)	46	0.88
AUA(I)	102	0.99	ACA(T)	98	1.30	AAA(K)	84	1.46	AGA(R)	32	1.34
AUG(M)	33	1.00	ACG(T)	22	0.29	AAG(K)	31	0.54	AGG(R)	27	1.13
GUU(V)	43	1.54	GCU(A)	46	0.80	GAU(D)	47	0.99	GGU(G)	21	0.56
GUC(V)	15	0.54	GCC(A)	98	1.70	GAC(D)	48	1.01	GGC(G)	34	0.91
GUA(V)	39	1.39	GCA(A)	77	1.34	GAA(E)	55	1.28	GGA(G)	56	1.49
GUG(V)	15	0.54	GCG(A)	9	0.16	GAG(E)	31	0.72	GGG(G)	39	1.04
G. yajiangensis											
Codon	Count	RSCU	Codon	Count	RSCU	Codon	Count	RSCU	Codon	Count	RSCU
UUU(F)	87	0.94	UCU(S)	59	1.10	UAU(Y)	103	1.14	UGU(C)	28	0.88
UUC(F)	98	1.06	UCC(S)	73	1.36	UAC(Y)	77	0.86	UGC(C)	36	1.13
UUA(L)	125	1.46	UCA(S)	92	1.72	UAA(*)	72	1.10	UGA(*)	66	1.01
UUG(L)	56	0.65	UCG(S)	17	0.32	UAG(*)	59	0.90	UGG(W)	27	1.00
CUU(L)	72	0.84	CCU(P)	63	0.95	CAU(H)	56	0.78	CGU(R)	19	0.72
CUC(L)	52	0.61	CCC(P)	91	1.37	CAC(H)	87	1.22	CGC(R)	20	0.76
CUA(L)	171	1.99	CCA(P)	89	1.34	CAA(Q)	92	1.34	CGA(R)	36	1.37
CUG(L)	39	0.45	CCG(P)	23	0.35	CAG(Q)	45	0.66	CGG(R)	19	0.72
AUU(I)	139	1.30	ACU(T)	76	1.02	AAU(N)	97	1.03	AGU(S)	33	0.62
AUC(I)	86	0.80	ACC(T)	100	1.34	AAC(N)	91	0.97	AGC(S)	47	0.88
AUA(I)	97	0.90	ACA(T)	102	1.37	AAA(K)	86	1.52	AGA(R)	35	1.33
AUG(M)	35	1.00	ACG(T)	20	0.27	AAG(K)	27	0.48	AGG(R)	29	1.10
GUU(V)	37	1.35	GCU(A)	44	0.81	GAU(D)	48	1.03	GGU(G)	15	0.40
GUC(V)	14	0.51	GCC(A)	93	1.72	GAC(D)	45	0.97	GGC(G)	32	0.85

GUA(V)	44	1.60	GCA(A)	75	1.39	GAA(E)	54	1.29	GGA(G)	70	1.87
GUG(V)	15	0.55	GCG(A)	4	0.07	GAG(E)	30	0.71	GGG(G)	33	0.88

Table S6. The amino acid abundance in the 13 PCGs of 16 *Garra* mitogenomes.

**Amino Acid**	***G. manipurensis***	***G. congoensis***	***G. dengba***	***G. flavatra***	***G. kempi***	***G. lamta***	***G. motuoensis***	***G. orientalis***
Ala	142	118	238	177	215	128	217	147
Arg	180	209	162	215	158	215	157	224
Asn	228	213	179	240	188	207	181	205
Asp	67	75	97	46	93	70	93	74
Cys	93	86	69	103	64	76	65	76
Gln	115	144	126	85	137	143	127	143
Glu	77	64	82	62	85	75	84	71
Gly	116	105	158	139	149	100	152	109
His	127	155	130	130	143	154	140	149
Ile	307	291	303	387	322	270	329	250
Leu	485	477	515	458	515	472	525	477
Lys	110	112	108	116	113	106	109	107
Met	60	65	38	40	36	55	35	58
Phe	194	160	182	191	185	149	189	144
Pro	284	296	286	272	266	322	263	336
Ser	313	365	307	311	321	375	320	363
Thr	322	310	290	328	298	328	285	322
Trp	51	44	37	51	27	39	25	42
Tyr	223	204	192	200	180	209	185	204
Val	91	77	116	106	110	74	113	78
Stp	214	232	186	142	197	235	208	222
**Amino Acid**	***G. poecilura***	***G. qiaojiensis***	***G. rufa***	***G. salweenica***	***G. spilota***	***G. tengchongensis***	***G. tibetana***	***G. yajiangensis***
Ala	202	218	142	218	131	222	230	216
Arg	207	158	207	162	235	164	143	158
Asn	234	190	199	187	215	193	180	188

Asp	55	91	70	90	72	90	95	93
Cys	110	62	92	63	71	66	73	64
Gln	83	132	144	132	136	131	132	137
Glu	62	90	70	78	74	86	86	84
Gly	154	146	117	161	95	151	150	150
His	142	145	152	146	165	146	145	143
Ile	344	328	265	298	267	330	310	322
Leu	483	533	478	537	459	522	522	515
Lys	118	108	104	119	110	110	115	113
Met	38	34	59	37	56	31	33	35
Phe	172	180	169	175	148	193	179	185
Pro	251	265	306	261	325	259	272	266
Ser	321	316	355	308	371	297	314	321
Thr	332	279	312	303	334	282	301	298
Trp	57	25	53	30	45	23	34	27
Tyr	188	187	195	177	192	179	184	180
Val	110	115	86	125	73	117	112	110
Stp	136	200	226	196	229	211	193	197

Table S7. The haplotype assignments of *G. manipurensis* inferred from partial mitochondrial COI gene sequences obtained from two localities in northeastern India. '*n*' indicates the number of sequences analyzed.

Locality	Number of Haplotype	*n*	Accession No.
Manipur	Hap_1	1	PZ247732
Mizoram	Hap_2	1	OQ269357
	Hap_3	2	OQ269356 and OQ269354
	Hap_4	1	OQ269355
